# Metaproteomics reveals insights into microbial structure, interactions, and dynamic regulation in defined communities as they respond to environmental disturbance

**DOI:** 10.1186/s12866-021-02370-4

**Published:** 2021-11-08

**Authors:** Him K. Shrestha, Manasa R. Appidi, Manuel I. Villalobos Solis, Jia Wang, Dana L. Carper, Leah Burdick, Dale A. Pelletier, Mitchel J. Doktycz, Robert L. Hettich, Paul E. Abraham

**Affiliations:** 1 Biosciences Division, Oak Ridge National Laboratory, 37831 Oak Ridge, Tennessee United States; 2 Department of Genome Science and Technology, University of Tennessee-Knoxville, 37996 Knoxville, Tennessee United States

**Keywords:** Rhizospheric microbiome, Microbial consortia, Defined community, Reductionist approach, Metaproteomics, PTMs

## Abstract

**Background:**

Microbe-microbe interactions between members of the plant rhizosphere are important but remain poorly understood. A more comprehensive understanding of the molecular mechanisms used by microbes to cooperate, compete, and persist has been challenging because of the complexity of natural ecosystems and the limited control over environmental factors. One strategy to address this challenge relies on studying complexity in a progressive manner, by first building a detailed understanding of relatively simple subsets of the community and then achieving high predictive power through combining different building blocks (e.g., hosts, community members) for different environments. Herein, we coupled this reductionist approach with high-resolution mass spectrometry-based metaproteomics to study molecular mechanisms driving community assembly, adaptation, and functionality for a defined community of ten taxonomically diverse bacterial members of *Populus deltoides* rhizosphere co-cultured either in a complex or defined medium.

**Results:**

Metaproteomics showed this defined community assembled into distinct microbiomes based on growth media that eventually exhibit composition and functional stability over time. The community grown in two different media showed variation in composition, yet both were dominated by only a few microbial strains. Proteome-wide interrogation provided detailed insights into the functional behavior of each dominant member as they adjust to changing community compositions and environments. The emergence and persistence of select microbes in these communities were driven by specialization in strategies including motility, antibiotic production, altered metabolism, and dormancy. Protein-level interrogation identified post-translational modifications that provided additional insights into regulatory mechanisms influencing microbial adaptation in the changing environments.

**Conclusions:**

This study provides high-resolution proteome-level insights into our understanding of microbe-microbe interactions and highlights specialized biological processes carried out by specific members of assembled microbiomes to compete and persist in changing environmental conditions. Emergent properties observed in these lower complexity communities can then be re-evaluated as more complex systems are studied and, when a particular property becomes less relevant, higher-order interactions can be identified.

**Supplementary Information:**

The online version contains supplementary material available at 10.1186/s12866-021-02370-4.

## Background

Similar to other eukaryotic organisms, plants have complex host-associated microbiomes that impact fitness and productivity. Microbial interactions within or outside plant tissues are intimately connected to essential processes including water and nutrient acquisition, stress response, and reducing disease or herbivory by priming host defenses [1–4]. Extensive research supports the co-evolution of binary interactions between plants and their associated microbes, yet the complex ecological interactions taking place in nature between microbes and the evolution of microbe-microbe interaction mechanisms remains poorly understood. Furthermore, it remains unclear as to what extent inter-microbial interactions shape microbial assemblages and function in nature and what molecular mechanisms are used by microbes to cooperate, compete, and persist in complex microbial consortia.

Inter-microbial interactions exist in various forms such as resource competition, synergism, antagonism, and these interactions can be altered through the environment. To fully understand the microbial dynamics in its environment, it is crucial to decipher these diverse and dynamic interactions and predict their competitive and cooperative potentials. Several computational and mathematical modeling approaches have been developed to predict the functional contribution of individual microbes in a community including their metabolic functions, inter-species interactions, and community dynamics. Generally, modeling approaches utilize ordinary differential equations (ODEs), annotated genomes and sequence read abundances [5]. Generalized Lotka–Volterra (gLV) models based on ordinary differential equations have been widely used in understanding temporal dynamics of microbial interactions. Compositional Lotka-Volterra (cLV), a recent non-linear modeling approach based on relative abundances is shown to accurately predict microbial trajectories over time in a community [6]. Constraint based genome-scale metabolic models are used in predicting the extent of resource competition and microbial metabolic interactions, which is facilitated by flux balance analysis (FBA) [7]. Besides these approaches, data-driven inferences of microbial interaction networks, such as ecology guided models that predict the metabolite cross-feeding interactions based on metagenomic and metabolomic measurements, are also valuable in understanding the community dynamics [8]. Taken together, microbial modeling approaches provide a guide to start understanding the functional state of microbial communities, as well as of their competitive and cooperative metabolic interactions. However, despite the usefulness of these tools, the key to fully understand the dynamics of microbial communities and the underlying principles within them, is to integrate predictive models with experimental data such as those obtained from experiments using defined microbial consortia [5–8].

In general, there are two complementary methodological frameworks to study microbial communities: holistically in natural environments or in laboratory-controlled delineated conditions. Both approaches have led to important discoveries in plant microbiome research [9]. Using the holistic approach to study the total rhizosphere proteome can provide fundamental information on environment-plant–microorganism interactions, however soil protein extraction is extremely challenging due to the complexity of soil matrix which led to low quantity and quality of the extracted proteins [10]. On the other hand, by first building a detailed understanding of relatively simple subsets of a microbial community, emergent properties that dictate microbial composition and function can be tested in more complex systems by adding different building blocks (e.g., hosts, community members) for different environments (e.g., liquid, soil). As more complex systems are studied, certain principles are expected to become less relevant, and these realizations begin to uncover higher-order interactions.

Cultivated microbes assembled into defined communities offer a definable landscape to explore microbe-microbe interactions and microbial mechanism and, when coupled to omics-based technologies, presents a powerful approach to achieve higher predictive power for genotype-to-phenotype associations. Acquiring high-resolution, quantitative data reflecting *in situ* conditions is a crucial starting step to understand microbial community dynamics. Estimation of microbial community dynamics has most commonly been generated by 16 S rRNA gene amplicon sequencing; however, these approaches have inherent limitations [11]. Microbial community members experience active vs. dormancy dynamics in their environment. Typically, at any given point in time, only a subset of microbial members is active, while others are in a state of dormancy and behave with strongly reduced metabolic rates [12, 13]. It is important to distinguish between active and inactive microbial taxa to understand their functional contributions to ecosystems. Therefore, pairing both 16 S rRNA transcript and 16 S rRNA gene sequencing attempts to address this issue by normalizing the measured rRNA levels by the abundances of individual members [13]. However, given that rRNA content or RNA/DNA ratios and growth rates do not always correlate, researchers has suggested that 16 S rRNA/rRNA gene sequencing is best interpreted as potential microbial activity [13].

Metaproteomics has been demonstrated to be a powerful method for accurate estimation of biomass from viable and functioning cells [14, 15]. Additionally, metaproteomics allows the large-scale identification and quantification of proteins from microbial communities which helps to characterize microbial membership, their functional roles and interspecies interactions in the community [15]. Furthermore, recent advancements in optimized bioinformatic pipelines for metaproteomics exploiting *de novo* peptide sequencing facilitate the identification and characterization of post-translational modifications (PTMs) in proteins, which provides unique insights into a largely unexplored level of microbial regulation/adaptation [16].

Herein, the objective of this study is to integrate a reductionist approach with high-resolution metaproteomics to study microbe-microbe interactions during the assembly of ten taxonomically diverse bacterial members frequently observed in the rhizosphere of *Populus* species. The assemblage process is interrogated for the 10-member microbial consortia co-cultured in a defined (MOPS + glucose) or a complex (R2A) growth medium. For this defined community (DefCom), we aim to (i) quantify changes in relative microbial population sizes for the community when passaged in different growth environments, (ii) characterize changes in microbial proteomes during the formation of stable communities, and (iii) reveal molecular mechanisms driving the community’s assembly and structure.

## Results

### Metaproteomics reveals details of microbial community stabilization and population equilibration as a function of growth media

**Fig. 1:**
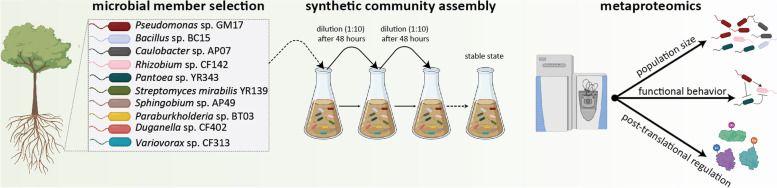
Schematic diagram of study design. Microbial members were selected based on phyla level abundance and tax diversity from natural *P. deltoides* (Pd) rhizosphere communities. These members were inoculated together and diluted 1:10 after 48 hours for 15 passages. Samples were analyzed by high-resolution metaproteomics to understand the community size and structure, molecular mechanisms underpinning microbial behavior and functions, and post-translational regulations. Created with BioRender.com

The ten diverse strains used in DefCom originated from roots of *Populus deltoides* [17–20] (Supplemental Fig. 1 A). The 10-member DefCom co-culture was grown in liquid defined media (MOPS + 0.2 % glucose) and complex media (R2A) subjected to growth/dilution cycles every two days until the community reached a stable state (state with minimal fluctuation of microbial community composition and abundance) (Fig. 1). These two media were selected to determine the effect of environmental filtering based on a single carbon source with limited essential nutrients and a more complex media with increased carbon resources and nutrients designed for long incubation periods.

**Fig. 2:**
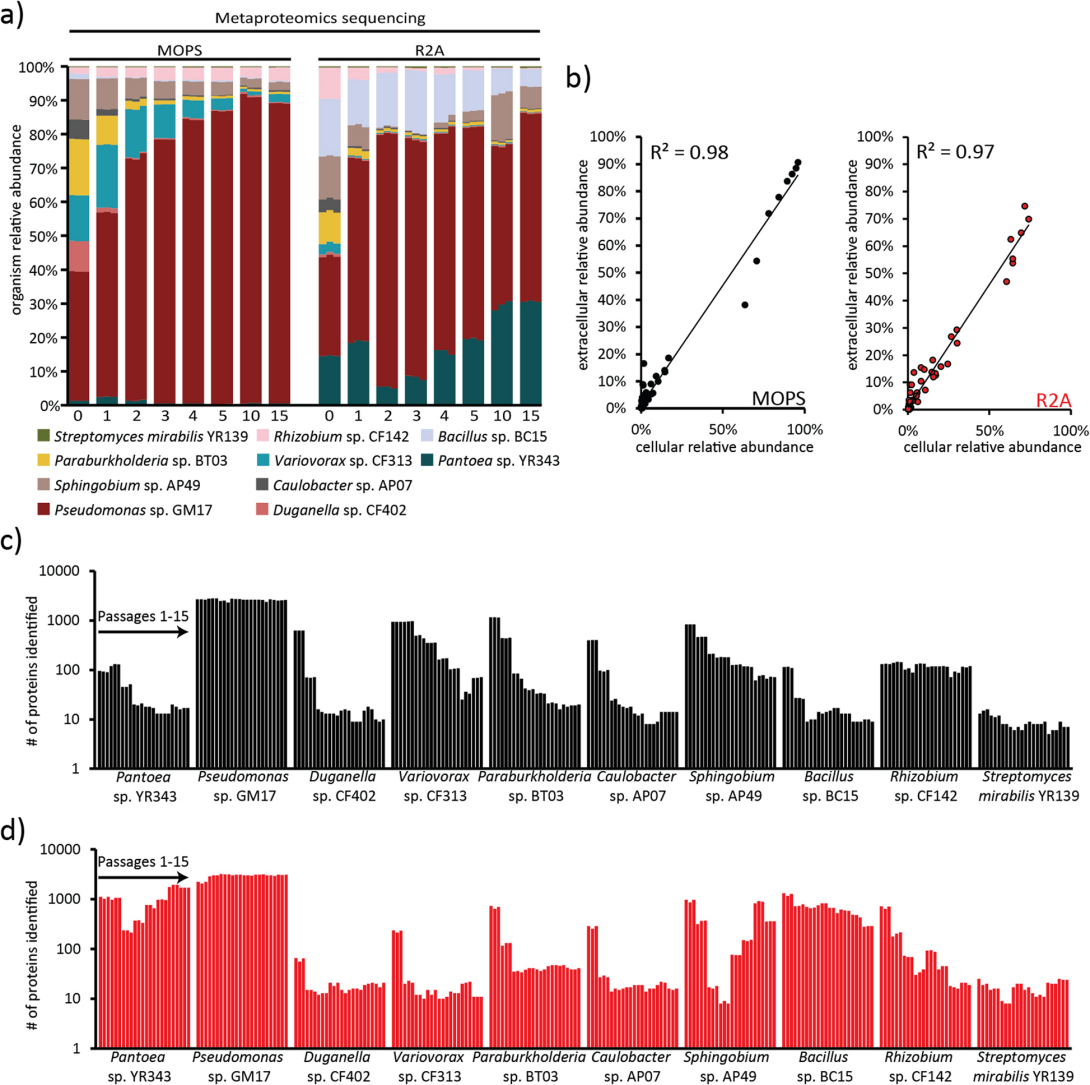
Assessment of microbial population size. (**A**) Microbial community composition from cell pellets using metaproteomics was estimated by total protein count for each community passaged in defined media MOPS + glucose (MOPS) or complex (R2A) media. There was a total of 15 passages per medium and 3 biological replicates per passage. Individual colors in the stacked bar charts represent each microbial member. (**B**) Cellular estimates of organism relative abundance plotted against extracellular estimates of organism abundance for each passage for MOPS (black circles) and R2A (red circles). Each circle represents the averaged abundance across replicates for a single passage. Proteome depth (number of proteins identified) is plotted for each microbe per sample measured in the defined microbial community passaged across **(C)** defined MOPS + glucose (black) and **(D)** complex R2A (red) media

Metaproteomic measurements of microbial communities provide a robust and accurate assessment of microbial cell population sizes [14] because proteins make up 40-60 % of bacterial cell biomass [21] and are known to have a linear correlation with cellular mass and volume [22]. Relative organism cell population size estimates, as determined by summed protein counts or protein summed abundances, shows that communities stabilized, reaching stable population equilibria in both growth media, but formed distinct population sizes and community structures between the two different growth media (Fig. 2 A and Supplemental Fig. 1B). Interestingly, three discrete community transition phases were observed in R2A media, in which the relative abundance and microbial membership were fluctuating systematically before reaching a stable state. A principal component analysis (PCA) for the metaproteomics dataset supports the observed discrete population changes for R2A media by revealing three major clusters (R0, R1 to R5 and R10 to R15) over the experimental passages (Supplemental Fig. 2 A). In the defined media, the 10-member community only experienced two discrete phases, with the community stabilizing after the second passage (Supplemental Fig. 2B).

Organism relative compositions were additionally assessed for extracellular fractions (Supplemental Fig. 1 C). The extracellular fraction, though most commonly used to characterize proteins secreted from cells, has a composition notorious for its abundance of proteins resulting from cell lysis (Supplemental Fig. 1D), and therefore provides a useful perspective for detecting recent mortality events. In general, organism relative abundances were highly similar between cellular and extracellular fractions (Fig. 2B). It can be reasoned that the measured correlation between these two fractions indicates that mortality and growth rates for each member in the community are relatively stable at the 48-hour sampling point.

### Quantifying microbial species reveals functional behaviors during community assembly

The ability to holistically study inter-microbial interactions and identify molecular mechanisms used by microbes to cooperate, compete, and persist in complex microbial consortia arguably requires expansive proteome coverage. Using state-of-the-art techniques, proteome coverage for the 10-member DefCom varied between microbes, ranging from tens to thousands of proteins per organism (Fig. 2B C) (Supplemental Tables 1 and 2). While the measured proteome depth can be affected by growth of microbes and metabolic rate, microbes often adapt their behavior to states of lower metabolic activity during unfavorable growth conditions [12]. As such, valuation of the completeness of a metaproteome measurement is not trivial and should not be used as a filter for interpretation.

To identify how members of this DefCom are responding to changes in the environment, significantly changing proteins were identified per organism using Student’s t-test between consecutive passages for both media. In complex media, three organisms, *Pseudomonas* sp. GM17, *Pantoea* sp. YR343, *Bacillus* sp. BC15, showed major proteome abundance changes across the passages (Supplemental Fig. 3). In minimal medium, *Pseudomonas* sp. GM17 was the only organism to show major proteome abundance changes. Given these observations, *Pseudomonas* sp. GM17, *Pantoea* sp. YR343, *Bacillus* sp. BC15 were further interrogated to identify the proteome-level changes in biological processes that influenced their functional behaviors and abundances in the community.

### *Pseudomonas* sp. GM17 behaves antagonistically during the community selection process

**Fig. 3:**
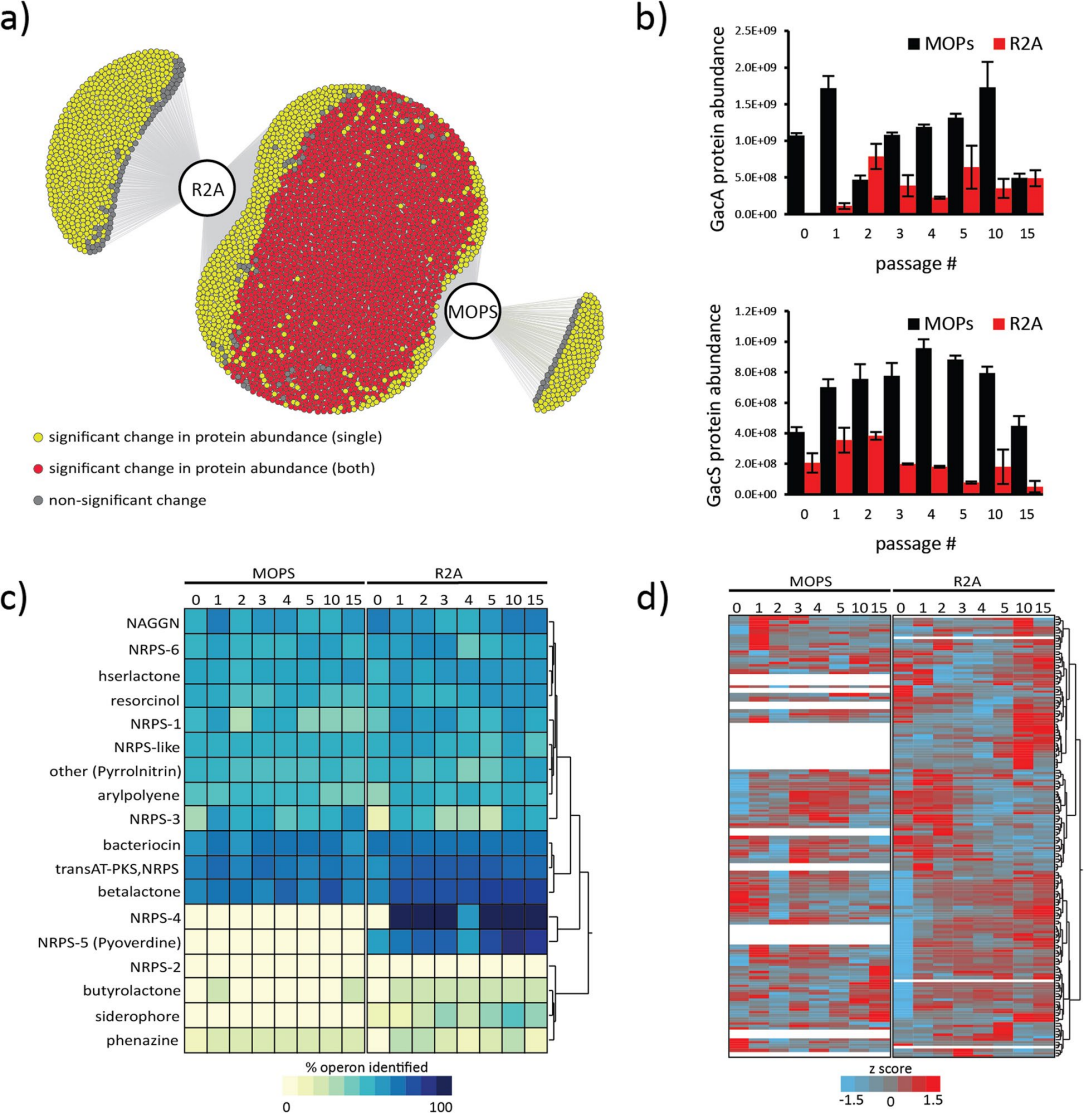
Metaproteomics analysis of *Pseudomonas* sp. GM17 functional behavior during community assembly. (**A**) Overlap of proteins identified between both media. Each node represents a unique protein accession, and the color indicates whether the relative protein abundance changed significantly based on ANOVA in one (yellow) or both media (red) or not significant in either (grey). Figure generated using DiVenn 2.0. (**B**) Relative protein abundance for the GacS sensor histidine kinase and the GacA response regulator in MOPS (black) or R2A media (red). Error bars represent standard error for each set of biological triplicates. (**C**) Heatmap (one-way clustering using ward method) illustration for 18 antibiotic and secondary metabolite gene clusters predicted by antiSMASH v5.0. Color gradient represents the percentage of proteins identified for a given gene cluster. (**D**). Heatmap (one-way clustering using ward method) illustration of relative protein abundances for proteins encoded by the 18 antibiotic and secondary metabolite gene clusters. Color gradient represents a standardized score calculated per protein and white represents proteins that were not quantified in a particular medium

*Pseudomonas* sp. GM17 was the dominant microbial member in both R2A and MOPS media, with the highest number of significant proteins in the majority of the pairwise comparisons (Supplemental Fig. 3). The observed proteomes between two media conditions have substantial protein and functional overlap, with about 79 % of the proteins expressed in both media conditions and with 17 % uniquely identified in R2A and 5 % uniquely identified in MOPS (Fig. 3 A). Additionally, the cluster of orthologous groups (COG) based annotation of the proteins showed a similar distribution of functional categories in both media suggesting similarity in mechanisms facilitating its dominance (Supplemental Fig. 4 A).

In general, *Pseudomonas* species are known to possess a large repertoire of antibiotics and secondary metabolites (SMs) that can equip them with a fitness advantage in multi-species consortium [23]. A recent study has shown *Pseudomonas* sp. GM17 to have negative interactions with majority of the microbial members in this 10-member DefCom [24]. In fact, notable changes in relative protein abundances were identified for a sensor histidine kinase GacS (WP_007927747.1), which plays a role in sensing environmental signals and GacA (WP_020295157.1), a response regulator activated by GacS (Fig. 3B). This two-component system is well known for its regulation of biosynthetic gene clusters (BGCs) and is involved in the biosynthesis of antibiotics and SMs in *Pseudomonas*. Based on antiSMASH v5.0 predictions [25, 26], the *Pseudomonas* sp. GM17 genome is suspected to have 18 BGCs encoding for the antibiotics and SMs (Supplemental Table 3). Of these 18 BGC, proteomics analysis identified proteins from 17 BGC in R2A and 14 in MOPS media (Fig. 3 C). Several BGCs in both media are involved in the biosynthesis of antibiotics such as pyrrolnitrin, phenazine, and resorcinol. Pyrrolnitrin biosynthesis proteins are involved in converting tryptophan to pyrrolnitrin. Pyrrolnitrin has been demonstrated to have antimicrobial activity [27]. Similarly, broad-spectrum antibiotic phenazines are known to enhance the competitiveness of *Pseudomonas* and are involved in antagonistic activity [28]. Likewise, resorcinol is also well known for fungal antagonism, biofilm formation, and biocontrol activity in *Pseudomonas* [29].

Three BGCs were uniquely identified in R2A media, of which two were predicted to encode non-ribosomal peptide synthetase (NRPS) and the other encodes for a siderophore (Supplemental Fig. 4B). NRPS are multimodular enzymes that can produce products with diverse properties such as toxins, siderophores or antibiotics [30]. Protein-level interrogation of one of the uniquely predicted NRPS BGC suggests involvement in pyoverdine biosynthesis. Pyoverdine is the fluorescent green-yellowish pigment produced by *Pseudomonas* species and represents a key siderophore [31]. This siderophore is a very efficient iron scavenger and helps *Pseudomonas* species adapt to changing environments and niche colonization [32]. Siderophores, in addition to iron scavenging, are also involved in the formation of complexes with other metals, antagonizing plant root microbes or facilitating antimicrobial activity [33]. While the proteomics data show that *Pseudomonas* synthesize multiple antibiotics and SMs in both media, few antibiotic- and SM-related proteins were uniquely identified in R2A, which suggests this environment and community is more competitive against *Pseudomonas* sp. GM17 when compared to minimal media (Fig. 3 C and D). Interestingly, the relative abundance trends of proteins associated with the production of antibiotics and secondary metabolites also differed between media (Fig. 3D).

Taken together, the proteomics analysis of *Pseudomonas* shows an increase in antibiotics, siderophore, and secondary metabolite production during the community selection process, indicating that *Pseudomonas* sp. GM17 is involved in antagonizing other microbial members. Pairwise-microbe interaction screen results reinforced these antagonistic properties of *Pseudomonas* by showing a presence of a zone of inhibition for most members of the community (Supplemental Fig. 4 C).

### *Pantoea* sp. YR343 is effective at adapting to the changes in the community in R2A media

**Fig. 4:**
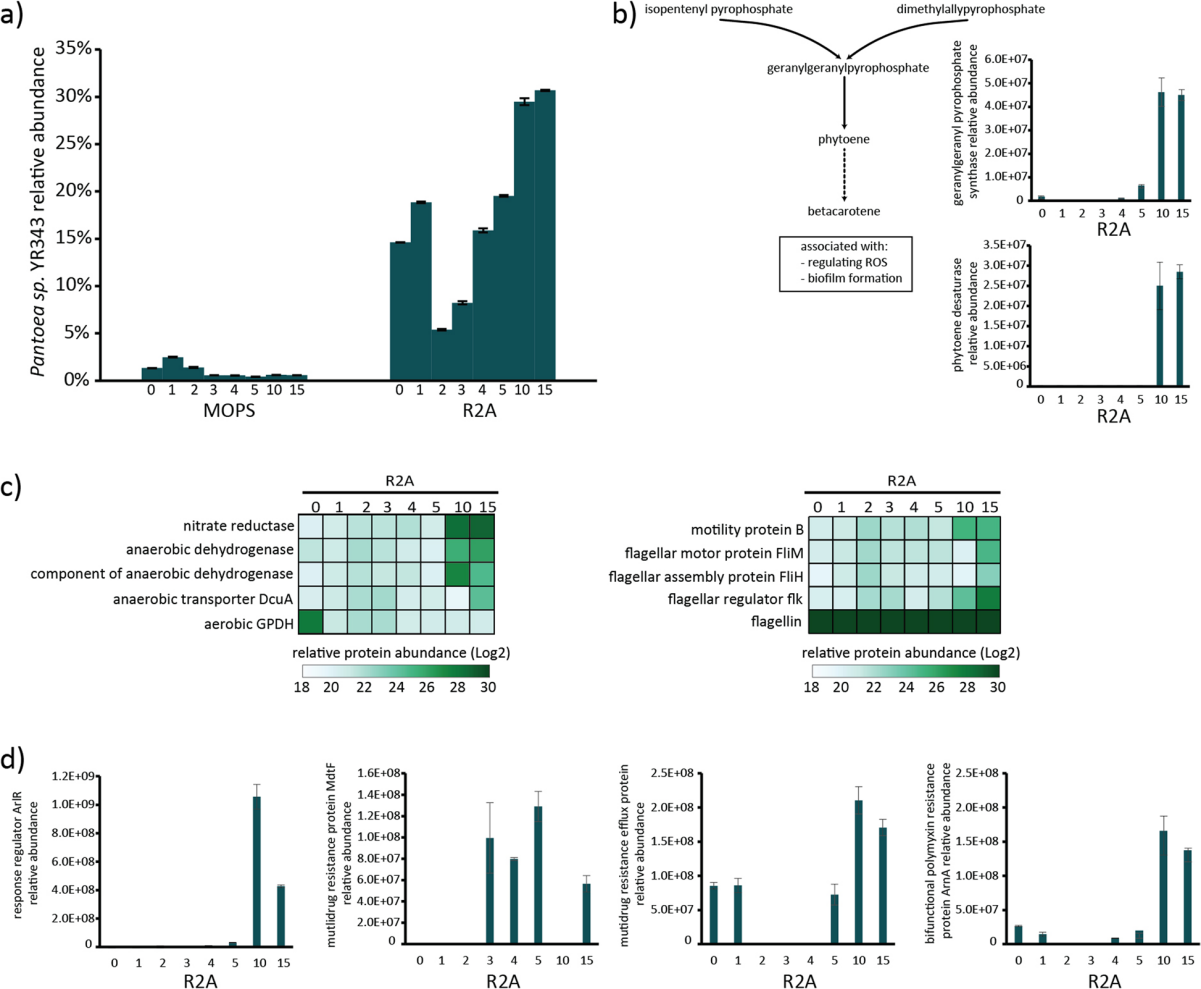
Metaproteomics analysis of *Pantoea* sp. YR343 functional behavior during community assembly. (**A**) Relative abundance of *Pantoea* organism abundance in MOPS and R2A media based on metaproteomics data. (**B**) Relative abundance across R2A passages for geranylgeranyl pyrophosphate synthase and phytoene desaturase, two key proteins involve in carotenoid biosynthesis. Error bars represent standard error for each set of biological triplicates. The dashed arrow in the flow diagram represents multiple steps in biosynthesis. (**C**) Heatmap of relative protein abundance for proteins involved in aerobic/anaerobic respiration and motility. (**D**) Relative abundances for proteins associated with defense responses to antagonist behaviors. Error bars represent standard error for each set of biological triplicates

In the minimal media, *Pantoea* sp. YR343 represent less than 1 % of stable community and most proteins identified are related to stress response (Fig. 4 A and Supplemental Fig. 5). Unlike its behavior in minimal media, *Pantoea* sp. YR343 population size changed substantially throughout the R2A passages, with a large reduction in size after passage 2 followed by a continuous gain in population size until representing ~31 % of the relative abundance in final stable community (Fig. 4 A). To understand how *Pantoea* sp. YR343 is adapting to the competitive or unfavorable environment and becoming a relatively abundant member of the community in R2A media, its respective proteome expression profile was extracted and analyzed (Supplemental Table 4).

Overall, *Pantoea* sp. YR343 was observed to effectively adapt to the changes in the community grown in R2A media by increasing the abundance of proteins related to stress responses, antibiotic resistance, motility, as well as shifting metabolism from aerobic to anaerobic processes. Increasing the relative abundances of proteins related to carotenoid biosynthesis, such as geranylgeranyl pyrophosphate synthase (J2V561) and phytoene desaturase (J2V5J0), helps to modulate membrane fluidity and aid in the *Pantoea*’s survival against oxidative stress, extremes pH, and toxins (Fig. 4B) [34–36]. Population size increase coincided with changes in chemotaxis and motility-related proteins; a behavior used to find environmental niches for optimal survival and growth. In an environment seemingly limited in oxygen, notable changes were observed in proteins related to anaerobic growth such as nitrate reductase (Accession ID: J3HM00, J3BZ11) and anaerobic dehydrogenase (Accession ID: J2VI80, J3HP12), suggesting *Pantoea* sp. YR343 survival benefited from an augmented metabolism (Fig. 4 C).

In response to the antagonistic behavior of *Pseudomonas* sp. GM17, *Pantoea* sp. YR343 effectively increased the abundance of several crucial defense proteins that protect against inhibition (Supplemental Fig. 4B). Relative protein abundances for several antibiotic/drug resistance proteins, such as multidrug resistance protein MdtF (J3HB42), response regulator ArlR (J3HGU6), bifunctional polymyxin resistance protein ArnA (J3BZA2) and multidrug resistance efflux pump (J3HPW6), increased across passages (Fig. 4D). Multidrug resistance protein MdtF and response regulator ArlR provide protection against a broad range of antibiotic compounds. Similarly, bifunctional polymyxin resistance protein ArnA helps in the resistance against polymyxin that breaks up the bacterial cell membrane and cationic antimicrobial peptides.

### *Bacillus* sp. BC15 sporulates as an adaptive response to nutritional competition

Based on protein content, *Bacillus* sp. BC15 was also a relatively abundant member in R2A media, but not in minimal media. Therefore, *Bacillus* sp. BC15 proteins were further investigated to identify which adaptive mechanisms were employed for these cells to thrive in R2A media (Supplemental Table 5).

**Fig. 5:**
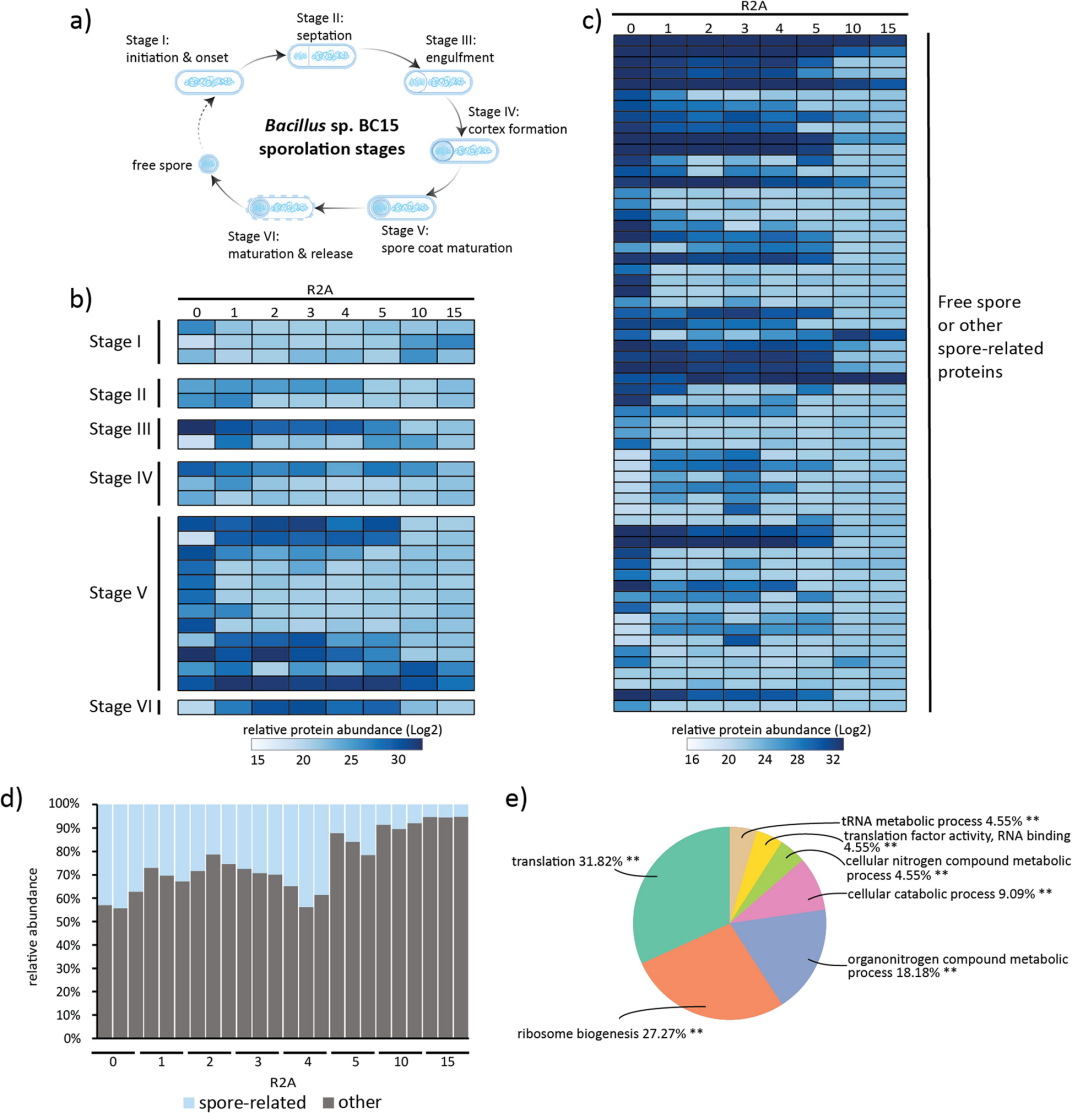
Metaproteomics analysis of *Bacillus* sp. BC15 functional behavior during community assembly. (**A**) Developmental stages of sporulation in *Bacillus* sp BC15. Under environmental stress or nutrient limited condition, *Bacillus* undergoes endospore formation which is accomplished across multiple stages of morphogenesis. Relative abundance of the proteins identified (**B**) across all stages and (**C**) free spores. Abundance shown are the average of three biological replicates. The color gradient represents the abundance in Log2 scale. (**D**)Proteome-wide relative abundance distribution of spore-related proteins compared to all other proteins. (**E**) The pie chart shows the enriched biological processes (p-value<0.05) of “all other” proteins. Gene ontology enrichment analysis was done using ClueGO

Using a rank-based distribution of *Bacillus* proteins based on abundance, spore-related functions were among the most abundant proteins observed (Supplemental Fig. 6). *Bacillus* species are known to cease growth and initiate sporulation under nutrient-limiting conditions and also in the presence of siderophores in the surrounding environment [37]. During sporulation, initially, an asymmetric cell division generates a smaller cell (forespore) and a larger cell (mother cell). The mother cell engulfs the forespore and mediates the development of the forespore into the spore through the production of the spore cortex and the inner and outer coat [37]. Upon spore maturation, the mother cell lyses, releasing the mature spore. This process is achieved in several developmental stages (Fig. 5 A). Metaproteomic results identified several *Bacillus* proteins from each stage of sporulation in R2A media, including stage 0 sporulation protein A (Spo0A; A0A1M7EFS7) (Fig. 5B) (Supplemental Table 5). Spo0A is a master transcription factor that binds to the promoters and regulates gene expression, driving the sporulation events [38]. Moreover, endospore proteins such as spore coat proteins (A0A1M7B6S6, A0A1M7DEY3 etc.), small acid-soluble proteins (A0A1M7C0T3, A0A1M6VBR5 etc.) were significantly upregulated in complex media (Fig. 5 C). Spore coat plays an important function in preventing spore degradation. Small acid-soluble proteins are found in the spore core and help to maintain the chromosomal DNA in a compact state [37].

Next, we assessed whether *Bacillus* sp. BC15 is becoming more or less metabolically active across the R2A passages. Overall, ~45 % of the *Bacillus* proteome expressed in early passages represents proteins associated with spores or sporulation processes (Fig. 5D) and this shifts to less than 10 % in passages 10 and 15. As the total abundance of spore-related proteins decrease with increasing passages, proteins associated with translation, ribosome biogenesis, and cellular metabolic and catabolic process increase in the final passages (Fig. 5E). In general, these results suggest that *Bacillus* sp. BC15 was not actively growing but rather existing in dormancy to survive during unfavorable conditions and persisted in the community by adapting differently until it could become more metabolically active, as represented by increased abundances in major cellular processes in the final passages.

### Post-translational modifications offer critical insights into how microbes are adapting to changing communities and environments

Post-translational modifications (PTMs) of proteins represents one of the most important yet understudied mechanism that microbes use to rapidly perceive and respond to changing conditions [39–42]. Recent advancements in metaproteomic experimental workflows now afford the ability to broadly study PTMs in bacterial isolates and communities [43–47]. Therefore, we further interrogated the data to demonstrate that the analysis of PTMs offers new insights into the function and behavior of individual microbial community members as they respond to changing communities and surrounding environments.

In general, PTMs were identified on ~40 % of proteins observed in this study (Supplemental Table 6) Modified proteins are expected to include both biologically relevant PTMs as well as those that are direct products of sample preparation procedures (e.g., carbamidomethylation on cysteine residues and methionine oxidation). Therefore, all observed PTMs were annotated as either a natural or artificial modification using a standardized nomenclature from the Unimod database as a guide (see Materials & Methods section) [48, 49]. Following this step, we observed that ~10 % of proteins identified in this study were proteins that contained biologically meaningful PTMs.

Amongst the biologically relevant PTMs observed in this study, the most frequently occurring types of PTMs were methylations, dehydrations, and oxidations/hydroxylations. Previous studies have shown these modifications to be quite abundant in proteomes from bacterial isolates as well as environmental communities [43, 45, 47]. The impact that these types of modifications have on proteins can be quite diverse but are frequently associated with altering protein-protein interactions or activity [50–52]. Based on annotations provided by cluster of orthologous groups (COG) categories, PTMs observed across this study impact many prominent cellular processes, including translation, ribosomal structure and biogenesis, energy production and conversion, protein turnover, and chaperon functions as well as amino acid metabolism and transport. Proteome-wide analysis of an abundant member of the community, such as *Pseudomonas* sp. GM17, suggests that changes in communities or the environment do not affect which types of PTMs are predominant or what cellular processes are being modified (Supplemental Figs. 7 and 8). Shifting our analysis from a proteome-wide to a protein-centric perspective allowed us to identify PTMs that occur at conserved residues in bacterial proteins (Supplemental Table 7) [39]. This examination revealed two types of modifications: proteins having static, seemingly always modified positions; and proteins with positions that are dynamic and reversible. Evidence for site-specific modifications that are static are interesting because they suggest a role critical to a protein’s primary function. In this study, one example is a β-methylthiolation modification in ribosomal protein S12 (Supplemental Fig. 9). This modification is localized on an aspartic acid residue universally conserved within bacteria and presumed to be structurally or functionally important because substitutions at this position are lethal [53–57]. All bacterial ribosomal S12 proteins sequenced across this entire study contained this modification, reinforcing previous findings that this modification is a static feature of the ribosome S12 protein. Beyond this well-known PTM, we also observed other proteins that have evidence for static modifications and warrant further interrogation to evaluate their essentialities (Supplemental Table 8).

**Fig. 6:**
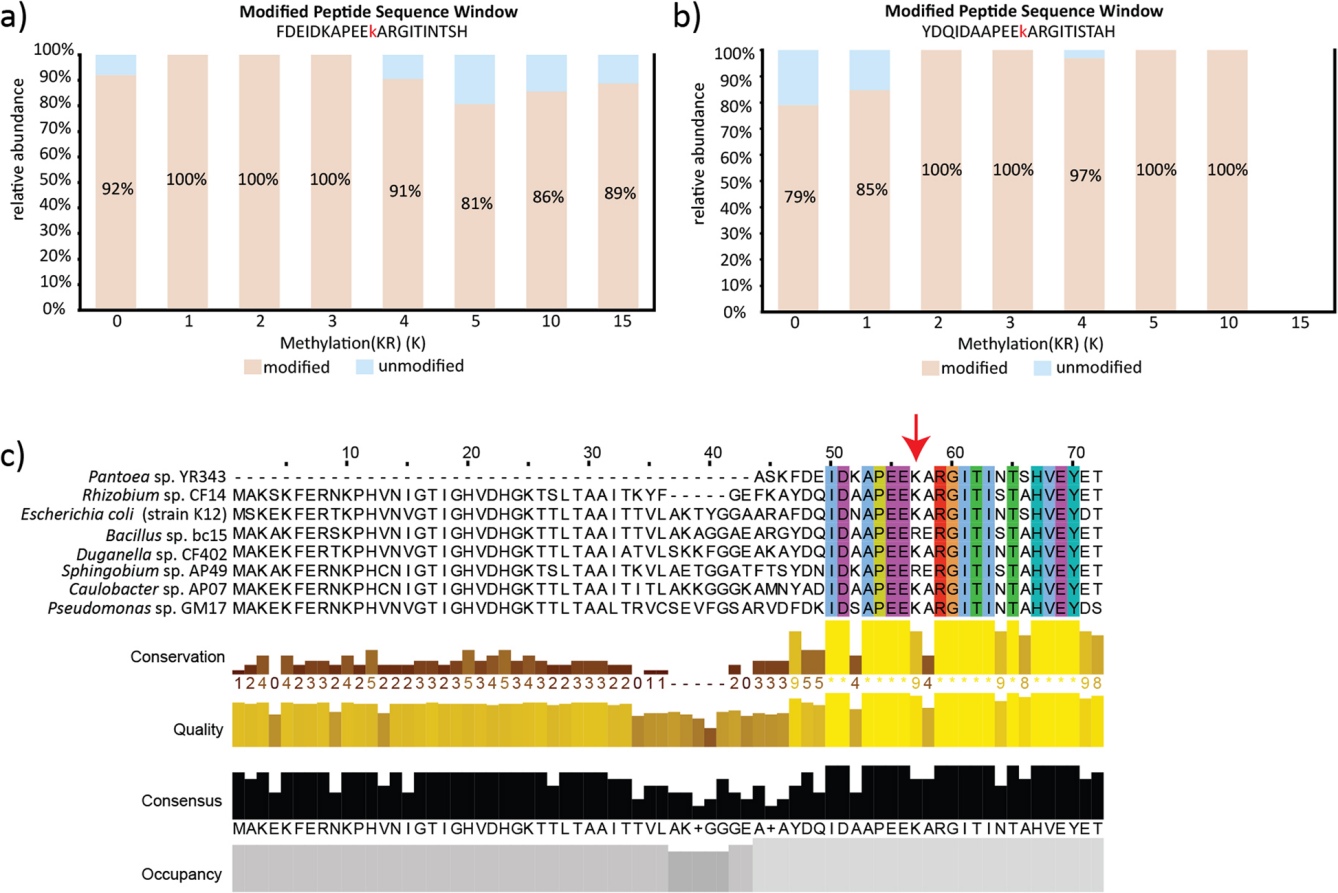
Dynamic regulation of methylation modification in lysine (K) residue of EF-Tu in nutrient-deprived organisms. Percentage abundances of K-methylated modified and unmodified peptides in the Elongation factor Tu proteins of (**A**) *Pantoea* sp. YR343 and (**B**) *Rhizobium* sp. CF142 in R2A media. (**C**) Multiple sequence alignment was performed for all Elongation Factor Tu proteins identified by metaproteomics in this study. The lysine amino acid position where the modification occurs in this study is highlighted with a red arrow. Unlike other organisms, *Bacillus* sp. BC15 and *Sphingobium* sp. AP49 have an arginine (R) residue at this position and no methylation modifications were observed for these protein sequences. For reference, the Elongation factor TU sequence of *E. coli* (strain K12) was added for comparison

Unlike static modifications, dynamic modifications are greatly influenced by experimental design and require adequate sampling to identify their roles. The identification and tracking of protein positions that are reversibly modified not only provides insights into a changing functional or structural state of a particular protein, but an extended understanding of how the organisms is perceiving or altering its behavior to changing conditions. For example, we observed a dynamic methylation on a lysine position in the elongation factor thermo unstable protein (EF-Tu) that could imply an organism is experiencing nutrient deprivation. EF-Tu proteins are one of the most conserved and abundant proteins expressed by bacteria [58, 59]. First reported in *E. coli*, the EF-Tu protein is hypermethylated at a lysine residue at position 56 under nutrient deprivation conditions and this modification is expected to reduce protein synthesis by affecting the ability of the protein to bind and hydrolase GTP [60–63]. Across this study, EF-Tu was predominately methylated at the conserved lysine position in *Pantoea* (Accession ID. J3HLV4) and *Rhizobium* (Accession ID. J1SLR3) (Fig. 6 A and B), however this modification was absent in EF-TU proteins observed in *Pseudomonas*, *Bacillus*, *Sphingobium*, *Caulobacter*, and *Duganella.* Based on the relative abundance of these microbes across different passages, we hypothesize that *Pseudomonas* maintains a relatively active protein synthesis rate across the entire experiment, whereas others like *Pantoea* and *Rhizobium* have a PTM-modified EF-Tu with reduced activity due to a lack nutrients. The lack of a modification in this protein for other members like *Bacillus* and *Sphingobium* is likely due to the presence of arginine residues at this position (Fig. 6 C), which seems to be the main reason behind the absence of this modification in some bacteria [62]. Although, it is possible that the EF-Tu methyltransferase does not function in these microbes. For other members like *Duganella* and *Caulobacter*, we believe that the detection limits of our instrumentation may preclude the observation of the methylated Lys residue, but we cannot completely discard the presence of this modification on their proteins.

## Discussion

In addition to plant-associated lifestyles, members of the rhizospheric microbiome adapt to local changes in environmental variables, such as resource availability, that shape the growth of individual populations and the interactions between species. Stochastic changes in the relative abundance of individual members have been demonstrated to have large downstream effects on community composition that can alter ecological dynamics [64]. The difference in stable microbial community composition observed in this study in different growth media could be due to the change in the chemical environment which largely affects the growth rate of microbial members. Interestingly, discrete community transition phases were observed across the passages in R2A media where the membership, as well as their relative abundance fluctuate suggesting the dynamic interaction between microbial members during the community assembly and selection process. Although this study provides the foundational understanding of rhizospheric-associated microbial community, dynamic interactions observed in the synthetic media may not be exact in the rhizospheric environment where factors like root exudates and other microbes effect the community assembly and selection process. Microbes interact with each other in a variety of ways, these interactions can be positive such as mutualism, synergism, and commensalism; or be negative such as antagonism, parasitism, predation, or competition [65]. Metaproteomics results allowed us to understand the microbe-microbe interactions in the DefCom and the molecular mechanisms driving the community level outcome.

Our metaproteomics analysis identified *Pseudomonas* sp. GM17 to be the dominant member of the community regardless of the chemical environment and environmental disturbances. The genus *Pseudomonas* are ubiquitous γ-proteobacteria well known for extreme versatility and adaptability [66]. The higher growth rate of *Pseudomonas* sp. GM17 in both media could have provided an initial competitive advantage that led to its dominance. Although *Pseudomonas* sp. GM17 was one of the fast-growing microbes, other microbes such as *Sphingobium* sp. AP49, *Paraburkholderia* sp. BT03 also has a similar growth rate [24]. However, these microbes did not represent a significant portion of the stable community. The proteome-wide analysis revealed that *Pseudomonas* expressed multiple proteins involved in the biosynthesis of siderophores that provide fitness advantage as well as antibiotics and secondary metabolites which are known to have biocontrol activity and facilitate antagonistic behavior. During antagonistic interaction the first population that produces inhibitory substances are unaffected or even gain a competitional advantage and survive in the habitat while other populations get inhibited. High growth rate as well as antagonistic behavior due to production of antibiotics and secondary metabolites helped in *Pseudomonas* sp. GM17 dominance by outcompeting or preventing the growth of other microbes [67].

As members of the community are challenged with unfavorable conditions created either intentionally by other members of the community (e.g., production of antibiotics from *Pseudomonas*) or unintentionally through the collective activity of community metabolism (e.g., limited nutrients, lower oxygen levels, etc.), microbial persistence requires the expression of necessary coping mechanisms. The proteome-wide analysis of *Pantoea* revealed a significantly higher proportion of proteins related to stress responses in minimal media but not necessarily of proteins related to coping mechanisms (Supplemental Fig. 5). Instead, in complex media, *Pantoea* sp. YR343 still experienced the stressful environment created by *Pseudomonas* and other members of the community but it was able to cope by increasing the abundance of proteins related to carotenoid biosynthesis, motility, and antibiotics resistance. Carotenoids are sterol analogs and studies have shown its ability to modulate membrane fluidity and role in the formation of membrane domains [68, 69]. Carotenoids aid in the survival of cells under harsh conditions, such as oxidative stress, extreme pH, and presence of toxins [34–36]. In *Pantoea* sp. YR343, carotenoid is required to regulate the sensitivity to reactive oxygen species, secretion, biofilm formation, and rhizosphere survival [70]. The defect in the gene involved in synthesizing carotenoids has been shown to affect the microbe’s growth, biofilm formation, and phytohormone production [70]. Similarly, motility directed by chemotaxis is an important means by which microbes avoid adverse conditions in their environment [71]. Microbes sense the presence of nutrients or other harmful chemicals in the environment with the help of chemotaxis related proteins which play an important role in environmental adaptation. Moreover, *Pantoea*’s ability to develop resistance against antibiotic and toxic compounds further helped to adapt in the changing environment. *Pantoea* is a facultative anaerobe that can grow in the presence and absence of oxygen, and our proteomics results also showed the shifting from aerobic to anaerobic respiration/metabolism as one of the coping mechanisms of *Pantoea* in R2A media.

While *Pantoea* effectively adapts to the changes in the environment of R2A media in the DefCom by increasing the abundance of proteins related to antibiotic resistance and motility as well as shifting metabolism from aerobic to anaerobic processes, *Bacillus* on the other hand, sporulated as an adaptive response. Endospores formed from the process of sporulation are morphologically distinct, metabolically dormant, and environmentally resistant, capable of surviving extreme environments [37, 72]. The identification of endospore proteins and sporulation proteins from various developmental stages implies that although *Bacillus* sp. has been identified as a key member of a stable community in R2A media, it is experiencing severe nutrient stress. A recent study has shown that siderophores can also act as interspecies cues that alter cellular development and accelerate sporulation in *Bacillus subtilis* [73]. Our results have shown that siderophore-related proteins from *Pseudomonas* were highly abundant, thus it is plausible that *Bacillus* sp. is sporulating in R2A media in the response of siderophores produced by the former.

Bacterial members within microbial communities adapt to their environmental conditions not only by modulating the abundance of the proteins they produce, but also by regulating the functions of these molecules. PTMs can modulate protein activity, conformation states, localization, and interactions. PTMs of proteins represents one of the most important, yet understudied mechanism that microbes use to rapidly perceive and respond to changing conditions [39–42]. PTM information allows an extended understanding of how an organism is perceiving or regulating its behavior to changing conditions. For example, the dynamic lysine methylation identified here in EF-Tu proteins has been suggested to reduce the rate of protein synthesis by affecting the ability of the protein to bind and hydrolase GTP under nutrient deprivation conditions [60–63]. Proteome-wide as well as protein-centric (i.e., static and dynamic) modifications identified in this study provide the proof of concept that optimized bioinformatics pipelines and high-resolution mass spectrometry not only affords the ability to broadly characterize PTMs in a biological system but also provides a level of sensitivity capable of revealing regulatory mechanisms influencing the activity of single proteins and we expect to continue mining this wealth of information to find novel and/or less-studied PTM regulatory mechanisms.

## Conclusions

The understanding of intricate ecological interactions between microbes, the extent to which inter-microbial interactions shape microbial community, and the comprehensive understanding of the mechanisms driving the microbe-microbe interaction represent key aspects of microbial ecosystem, yet these remain not well understood. The reductionist approach presented here provided an informative and useful opportunity to study naturally occurring complex microbial communities in a controlled and tractable laboratory setting. Together with high-resolution metaproteomics, an accurate assessment of microbial population sizes was obtained for this 10-member DefCom while simultaneously providing detailed understanding of proteome- and protein-level changes that help elucidate the biological process underpinning community assembly across two distinct growth conditions. By identifying building blocks of complex microbial behaviors that exist in defined communities, mechanisms underlying microbe-microbe offer some degree of prediction that can be further tested as more natural settings become integrated into experimental design. Moving forward, we envision data-rich metaproteomics datasets will become an integral source of information in modeling microbe-microbe interactions and a valuable datatype in predictive biology and discovery-based research environments, like The Department of Energy Systems Biology Knowledgebase (KBase) [74].

## Materials and methods

### Preparation of 10-member microbial communities.

Bacterial strains used in this study are *Populus deltoides* derived *Bacillus* sp. BC15, *Caulobacter* sp. AP07, *Duganella* sp. CF402, *Pantoea* sp. YR343, *Paraburkholderia* sp. BT03, *Pseudomonas* sp. GM17, *Rhizobium* sp. CF142, *Sphingobium* sp. AP49, *Streptomyces* sp. YR139, and *Variovorax* sp. CF313 [17, 20, 24, 70, 75–80] (Supplemental Fig. 1 A). The growth rates (OD_600nm_) for these 10 microbial strains has been previously measured in R2A and MOPS+ 0.2 % glucose media [24]. Equal volumes of all 10 isolates with the same normalized OD_600_ were mixed in 10 mL of R2A complex medium (Teknova, # R0005) [81] and 10 mL of MOPS minimal medium [82] supplemented with 0.2 % glucose at 30 °C with shaking at 200 rpm. The cultures were transferred into fresh media by diluting 1:10 every 48 h for a total of 15 passages. The remaining cultures were pelleted by centrifugation at 12,000 rpm for 15 min, and the spent culture supernatants were stored at -80 °C. Based on the 16 S rRNA gene amplicon sequencing results, 8-passages (0, 1, 2, 3, 4, 5, 10, 15, note: Passage 0 is after 48-hour growth in media) with three biological replicates for both R2A and MOPS+glucose were analyzed for proteomic analysis [24].

### Cellular protein extraction

Cell pellets were solubilized in 600 µL of lysis buffer (4 % sodium dodecyl sulfate (SDS) (Sigma-Aldrich, #L6026, USA) in 100 mM Tris, pH 8.0) supplemented with 1x Halt Phosphatase Inhibitor Cocktail (Thermo Scientific, #78,426, USA). Samples were vortexed and then further disrupted with a Bullet Blender storm 24 (Next Advance) for 5 min at setting #10 using 0.15mm Zirconium oxide beads (Next Advance, #ZROB015) at 3:1 sample to bead ratio. Samples were then placed in a heat-block for 10 min at 90 °C and centrifuged at maximum speed for 2 min to get rid of the foam. Protein concentration was measured using a Nanodrop One spectrophotometer (Thermo Scientific). Samples were centrifuged again at maximum speed for 10 min. The cell lysates were transferred into fresh Eppendorf tubes. Samples were reduced with 10mM dithiothreitol (DTT) (Sigma Life Science, #43,815, USA) and incubated at 90 °C for 10 min and then alkylated with 30 mM iodoacetamide (IAA) (Sigma Life Science, #I1149, USA) for 15 min in dark to prevent the reformation of disulfide bonds. In fresh tubes, Sera-Mag beads (GE Healthcare Life Sciences, #GE45152105050250, UK) were added at the 1:1 protein to beads ratio and proteins were extracted by protein aggregation capture method as described previously [83]. Beads were washed with acetonitrile (ACN), LC-MS grade (EMD Millipore Corp., #AX0156-1) on a magnetic rack, after removal from the magnetic rack, samples were added to tubes and then adjusted to 70 % ACN. At this point, proteins started to precipitate and bind to the beads. Samples were let to settle for 10 min. After 10 min, settled beads were gently resuspended. Samples were again let to settle for another 10 min. Samples were then placed on a magnetic rack. The supernatant was removed using a vacuum system. Samples were further washed with 1 mL ACN and 1mL of 70 % ethanol (EMD Millipore Corp., #EX0278-1) while on a magnetic rack. A total of 1mL volume of Tris buffer were added to the sample tubes with proteins bound to magnetic beads. The samples were removed from the magnetic rack. Proteins were digested with two separate and sequential aliquots of sequencing grade trypsin (Thermo Scientific, #90,057, USA) at 1:75 (wt/wt) protein:trypsin ratio for overnight, followed by 3 h at 37 °C at constant shaking. The samples were then adjusted to 0.1 % TFA (Sigma-Aldrich, #302,031, USA), vortexed, and centrifuged at max for 10 min. Vivaspin 500,10 kDa MW cutoff filters (Sartorius, #VS0102) were equilibrated with 500 µL of Tris buffer and centrifuged at 12,000 g for 15 min. After equilibrating, the samples were added to the Vivaspin columns and centrifuged at 12,000 g for 15 min. Tryptic peptides flow-through were collected and desalted on Pierce peptide desalting spin column (Thermo Scientific, #89,852, USA) as per the manufacturer’s instructions. Desalted peptides were vacuum dried with a SpeedVac Concentrator (Thermo Scientific) and then resolubilized in 0.1 % formic acid (Fisher Chemical, #A117-50). Peptide concentrations were measured using the Nanodrop instrument and transferred to the auto-sampler vials for LC-MS/MS measurement.

### Extracellular protein extraction

Spent media samples were adjusted to 2mM EDTA (Thermo Fisher Scientific, #R1021) on ice. The samples were filtered using Vivaspin 20mL Centrifugal Concentrators with 5000 Da molecular weight cut off (Sartorius, #VS2012) by spinning at 4,000 rpm at 4 °C for 30 min. The Vivaspin 5000 Da molecular weight cut-off filters were washed with 1 mL of 4 M urea (Sigma Life Science, #51,456, USA) in Tris buffer. The proteins were resuspended on the filter membrane with a final volume of 500 µL 4 M urea in Tris buffer. DTT was added to a final concentration of 10 mM for reduction of disulfide bonds and incubated samples at room temperature for 30 min. Samples were then alkylated by adding IAA to a final concentration of 30 mM and placed at room temperature in the dark for 15 min. Protein concentrations were measured by Nanodrop. Proteins were digested with Pierce Trypsin Protease, MS-grade at 1:75 wt/wt protein to trypsin ratio, overnight at 37 °C with constant shaking. Samples were diluted with Tris buffer to a final concentration of 2 M urea and a second 3 h digestion with trypsin was performed using the same conditions. The tryptic peptide flow throughs were collected by spinning at 4,000 rpm for 15 min. The samples were acidified with 0.5 % formic acid and then desalted using Pierce peptide desalting spin column as per the manufacturer instructions. Desalted peptides were vacuum-dried with a SpeedVac Concentrator and then resolubilized in 0.1 % formic acid. Peptide concentrations were measured using the Nanodrop instrument and transferred to auto-sampler vials for LC-MS/MS measurement.

### Protein identification and quantification

Each sample was analyzed using two-dimensional (2D) liquid chromatography (LC) on an Ultimate 3000 RSLCnano system (Thermo Fischer Scientific, USA) coupled with a Q Exactive Plus mass spectrometer (Thermo Fischer Scientific, USA). For each sample, an aliquot of digested peptide mixture was injected across an in-house built strong cation exchange (SCX) Luna trap column (5 μm, 150 μm X 50mm; Phenomenex, USA) followed by a nanoEase symmetry reverse phase (RP) C18 trap column (5 μm, 300 μm X 50mm; Waters, USA) and washed with an aqueous solvent. Cellular peptide mixtures were separated and analyzed across three successive SCX fractions of increasing concentrations of ammonium acetate (35mM, 50mM, and 500mM), each followed by a 100-minute organic gradient (250nL/min flow rate) to separate peptides across an in-house pulled nanospray emitter analytical column (75 μm X 350 mm) packed with Kinetex RP C18 resin (1.7 μm; Phenomenex, USA). The extracellular peptide mixtures were separated using the same separation regime, albeit with only one SCX fraction. All MS data were acquired with Thermo Xcalibur (version 4.2.47) using the topN method where N could be up to 10. Target values for the full scan MS spectra were 3 × 10^6^ charges in the 300 – 1,500 m/z range with a maximum injection time of 25 ms. Transient times corresponding to a resolution of 70,000 at m/z 200 were chosen. A 1.6 m/z isolation window and fragmentation of precursor ions were performed by higher-energy C-trap dissociation (HCD) with a normalized collision energy of 27 eV. MS/MS scans were performed at a resolution of 17,500 at m/z 200 with an ion target value of 1 × 10^5^ and a maximum injection time of 50 ms. Dynamic exclusion was set to 20 s to avoid repeated sequencing of peptides. All MS raw data files were analyzed using the Proteome Discoverer software (version 2.3, Thermo Fischer Scientific, USA). Each MS raw data file was processed by the SEQUEST HT database search algorithm [84] and confidence in peptide-to-spectrum (PSM) matching was evaluated by Percolator [85]. Peptide and PSMs were considered identified at *q*<0.01 and proteins were required to have at least one unique peptide sequence. Protein relative abundance values were calculated by summing together peptide extracted ion chromatograms.

Proteins with at least one unique peptide were exported from Proteome Discoverer. Protein abundances were log2-transformed, LOESS normalized and mean-centered across the entire dataset using InfernoRDN software [86]. From this normalized dataset, protein abundances subset for each microbe were extracted and further mean-centered by InfernoRDN. For this study, pairwise comparisons were performed across different passages (i.e., Passage 0 vs. Passage 1, Passage 1 vs. Passage 2, etc. for R2A and MOPS+glucose) to identify the differences between the passages. The analysis was limited to proteins that were identified in at least two out of three biological replicates of at least one sample to improve the robustness of the downstream analysis. The abundance values for proteins with missing values were imputed with random values drawn from the normal distribution (width 0.3, downshift 2.8) using Perseus software v.1.6.12.0 [87]. Student’s *t*-tests were performed to identify the differences in protein abundance values between different passages. Proteins are characterized as significantly differentially abundant if they pass the significance threshold of p-value ≤0.05 and absolute log2 fold-change difference greater than 1.

### PTM identification by PEAKS

Raw spectral data collected from both complex and minimal media sample sets were re-searched by *de novo*-assisted database searches against the 10-member community proteome accompanied with common contaminant proteins using PEAKS DB, PEAKS PTM, and PEAKS SPIDER in PEAKS X Studio (Bioinformatics Solutions, Waterloo, Canada). The peptide and fragment ion mass tolerances were set to ±10 ppm and ±0.02 Da, respectively. “Trypsin” was set as the enzyme parameter. Features associated with chimera scan were enabled. *De novo* ALC score was set at >90 %. A false discovery rate of 1 % was applied to accept the peptide sequences and a minimum of three peptides were required to identify a protein. For PEAKS DB, carbamidomethylation (+57.02) of cysteine was set as fixed modification and oxidation (+15.99) of methionine was set as a variable modification. The PEAKS PTM algorithm was used to identify other types of modifications by allowing the search against all possible modifications from the Unimod database [88]. Similarly, PEAKS SPIDER algorithm was used to detect any possible *de novo* sequencing errors and homology peptide mutations.

To report the number and percentages of modified proteins, only proteins with unique peptide sequences, that is, without any modifications, that were present in at least two out of three biological replicates per passage were considered. Peptide uniqueness was verified with the Protein Coverage Summarizer Tool (https://omics.pnl.gov/software/protein-coverage summarizer) and the data filtered using the Perseus software (http://www.perseus-framework.org) [87]. Unimod categories used to discriminate biologically relevant PTMs to those PTMs that can be the products of sample preparation/handling were: “Post-translational”, “Multiple”, “N-linked glycosylation”, and “O-linked glycosylation”.

The PTM profile tables reported by the PEAKS software were used to interrogate individual proteins and their PTMs. These tables list the summed abundance of modified and unmodified versions of unique and shared peptides that fall within a modified peptide sequence window in a protein. Only summed abundances reported in at least two out of three biological replicates per passage and conditions we considered. Summed abundance values were averaged per passage, and the percentage abundance ratios of modified to unmodified peptides were calculated from them. All PTM related figures presented with the manuscript were created with JMP Pro 14 (https://www.jmp.com/en_ca/software/predictive-analytics-software.html).

## Supplementary Information

**Additional file 1.**

**Additional file 2.**

**Additional file 3.**

**Additional file 4.**

**Additional file 5.**

**Additional file 6.**

**Additional file 7.**

**Additional file 8.**

**Additional file 9.**

## Data Availability

All proteomics spectral data in this study were deposited at the ProteomeXchange Consortium via the MASSIVE repository (https://massive.ucsd.edu/). The ProteomeXchange project identifier is PXD025747 and the data can be reviewed under the username “MSV000087344_reviewer” and password “PMI_microbiome”.

## Abbreviations

2D: Two-Dimensional
ACN: Acetonitrile
BGC: Biosynthetic Gene Clusters
cLV: Compositional Lotka-Volterra
COG: Cluster of Orthologous Groups
DefCom: Defined Community
DTT: Dithiothreitol
EF-Tu: Elongation Factor Thermo Unstable
FBA: Flux Balance Analysis
gLV: Generalized Lotka–Volterra
HCD: Higher-Energy C-Trap Dissociation
IAA: Iodoacetamide
KBase: Department of Energy Systems Biology Knowledgebase
LC: Liquid Chromatography
LOESS: Local regression
MOPS: Potassium morpholinopropane sulfonate
MS: Mass spectrometry
NRPS: Non-Ribosomal Peptide Synthetase
OD: Optical Density
ODE: Ordinary Differential Equations
PCA: Principal Component Analysis
Pd: Populus deltoides
PSM: Peptide-spectrum match
PTM: Post-Translational Modifications
R2A: Reasoner's 2A media
RP: Reverse Phase
SCX: Strong Cation Exchange
SDS: Sodium Dodecyl Sulfate
SM: Secondary Metabolites
Spo0A: Stage 0 Sporulation Protein A
