# Supplementary material for: Metaproteomics reveals insights into microbial structure, interactions, and dynamic regulation in defined communities as they respond to environmental disturbance
Source: BMC Microbiol. 2021 Nov 8;21:308. doi: 10.1186/s12866-021-02370-4 (PMC8574000; doi:10.1186/s12866-021-02370-4)
Supplement: Supplementary file 1 — Additional file 1. [file 12866_2021_2370_MOESM1_ESM.docx]

Supplementary Figures

**Metaproteomics reveals insights into microbial structure, interactions, and dynamic regulation in defined communities as they respond to environmental disturbance**

Him K. Shrestha^1,2,^ *, Manasa R. Appidi^1,2, *^, Manuel I. Villalobos Solis^1^, Jia Wang^1^, Dana L. Carper^1^, Leah Burdick^1^, Dale A. Pelletier^1^, Mitchel J. Doktycz^1^, Robert L. Hettich^1^, and Paul E. Abraham^1, ++^

^1^Biosciences Division, Oak Ridge National Laboratory, Oak Ridge, Tennessee 37831, United States.

^2^Department of Genome Science and Technology, University of Tennessee-Knoxville, Knoxville, Tennessee 37996, United States.

* Authors contributed equally

^++^ **Corresponding author:** [abrahampe@ornl.gov](mailto:abrahampe@ornl.gov) (Paul E. Abraham)


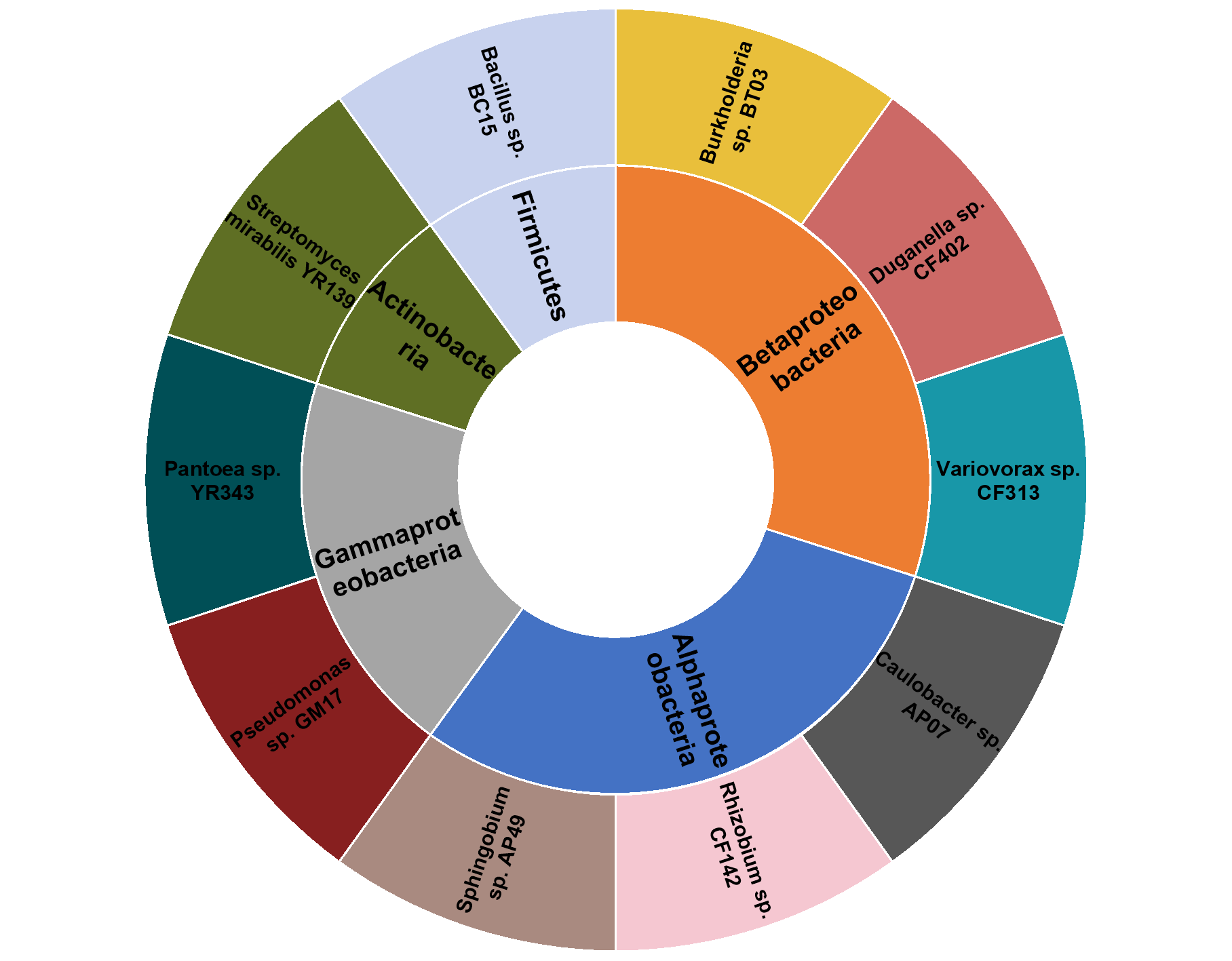

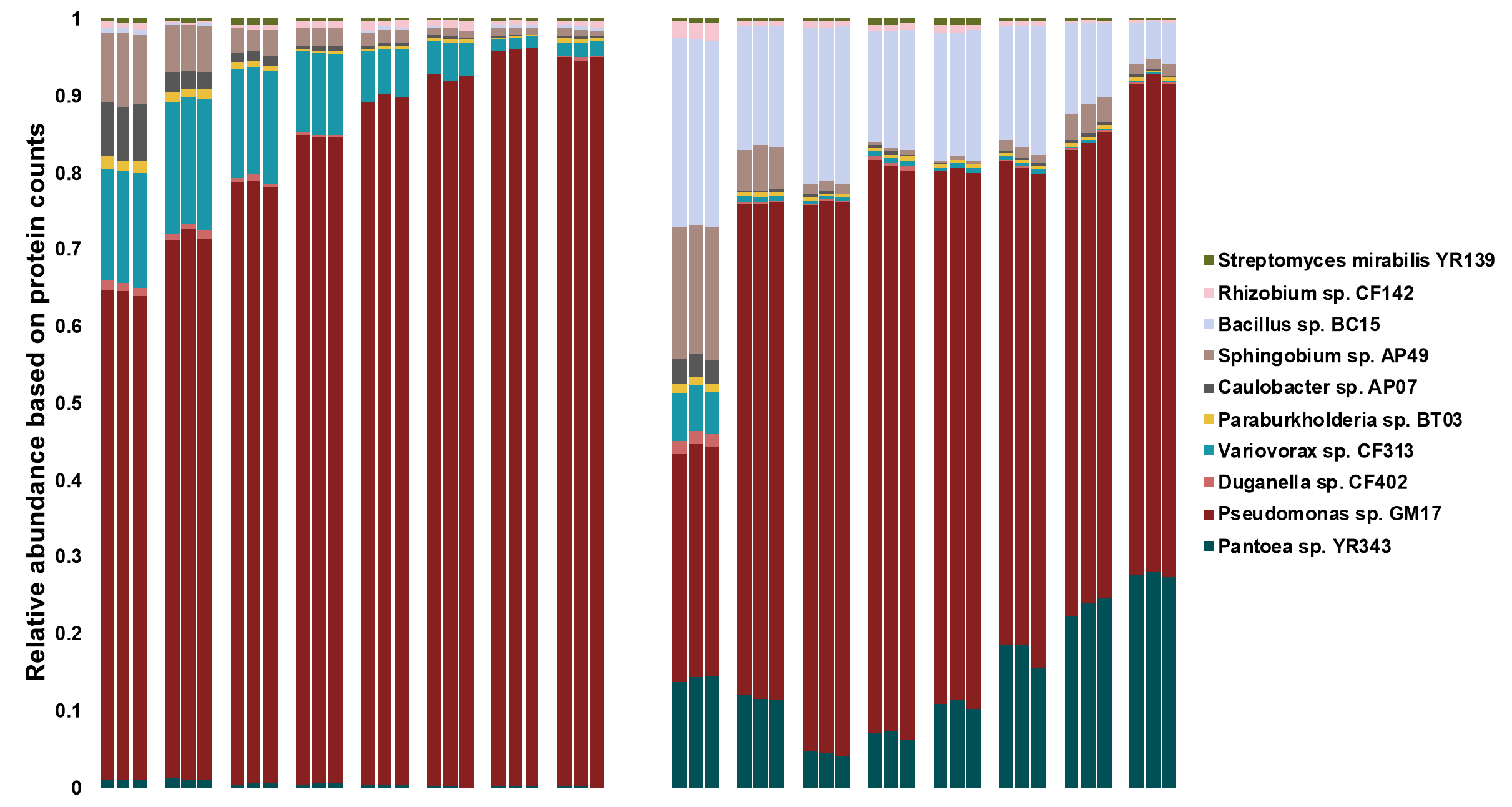

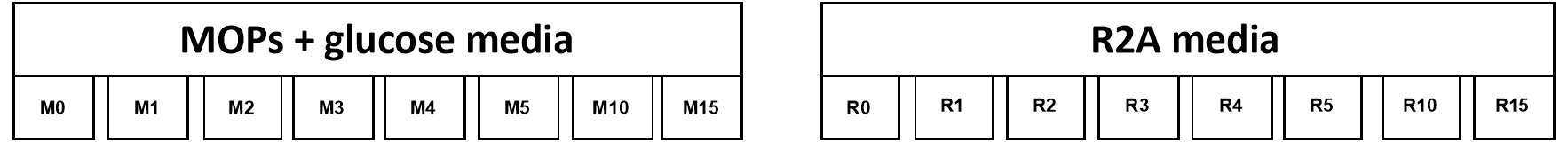


**(b)**

**(c)**

**(a)**

**(d)**


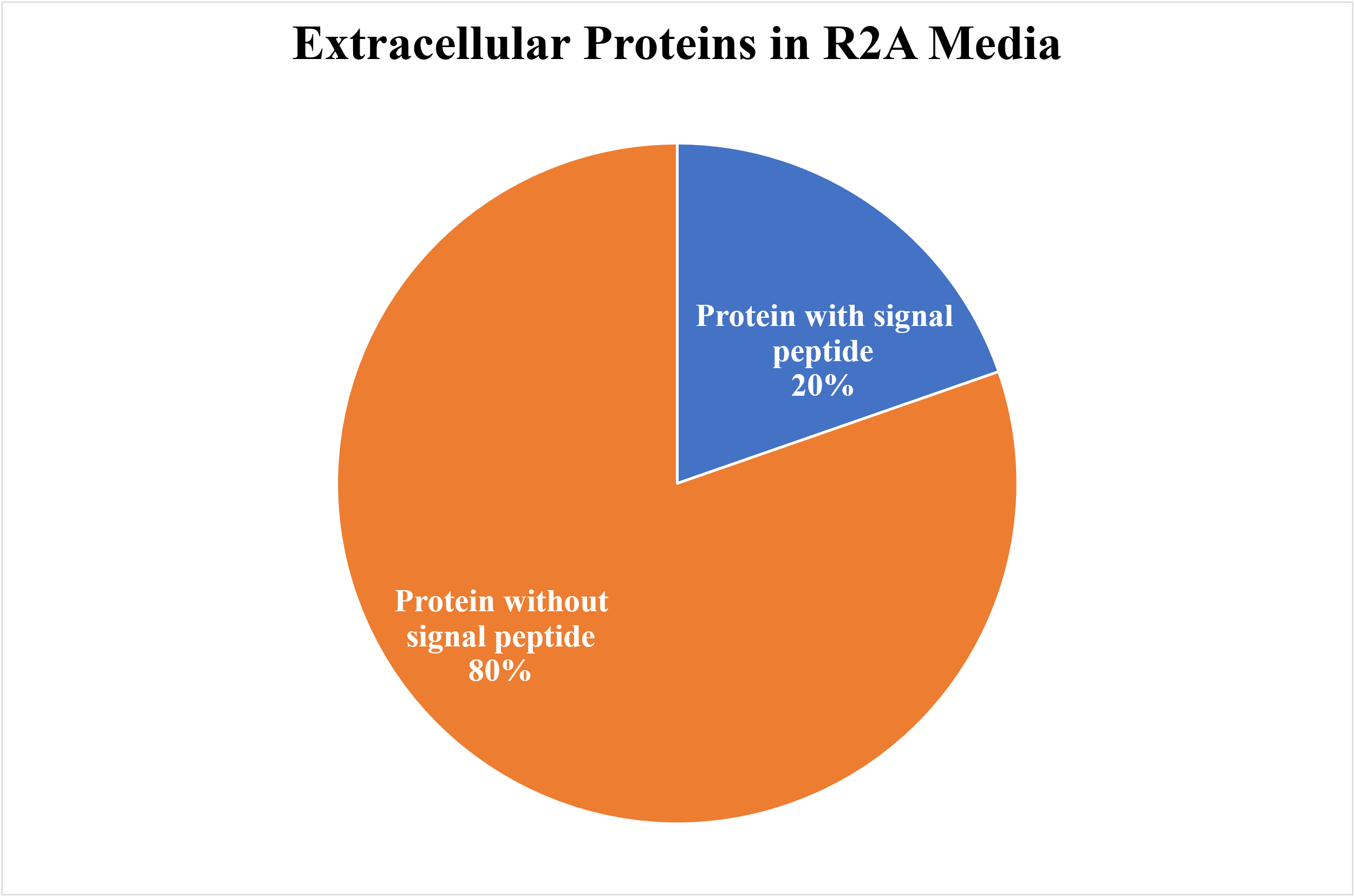

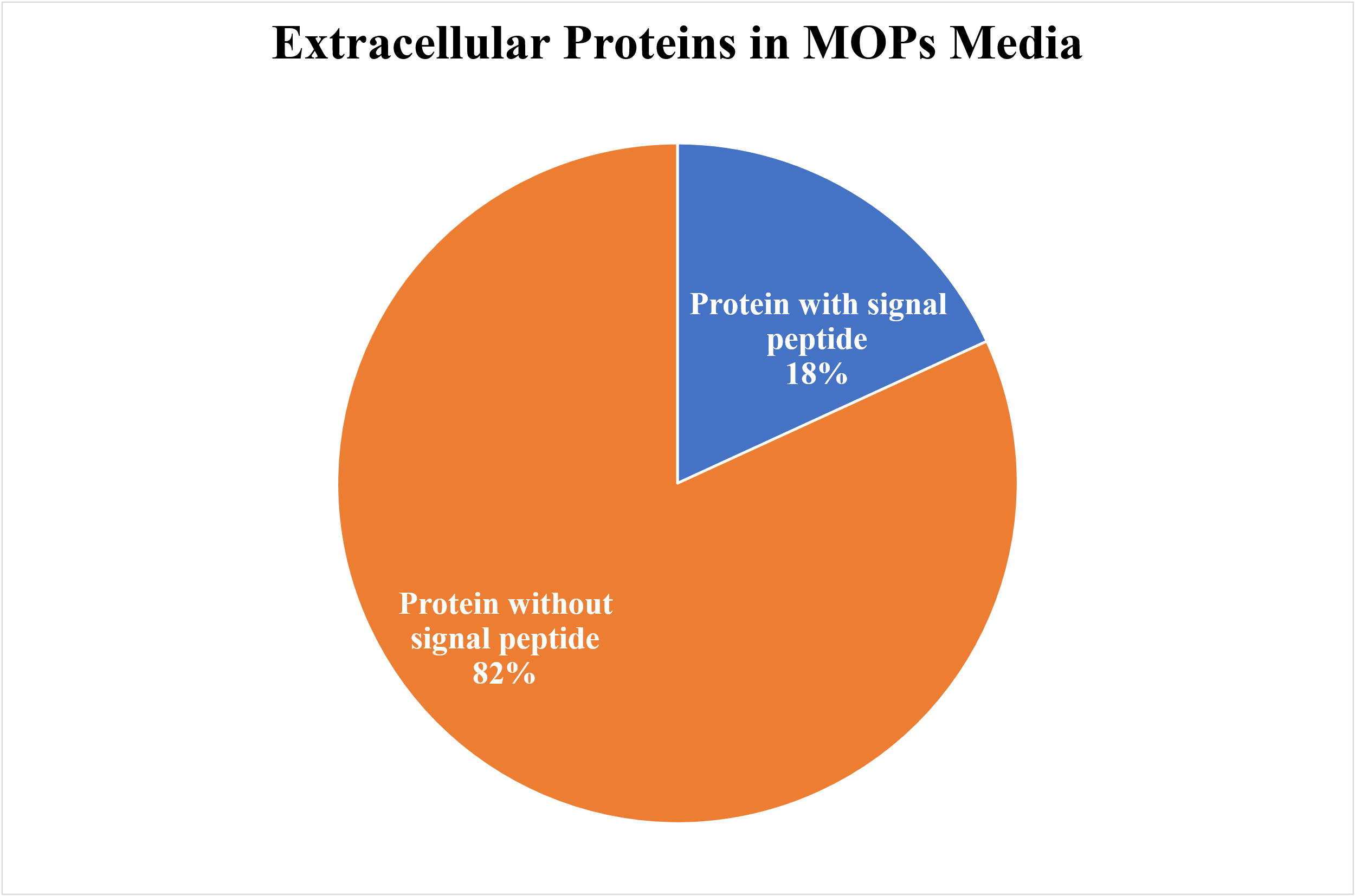


**Supplemental** **figure** **1:** **Assessment** **of** **defined** **microbial** **community.** **(A)** Community members were selected based on phyla level abundance to represent natural *P.* *deltoides* (Pd) communities. Inner doughnut plot shows the phyla level distribution and outer doughnut shows the individual members chosen for the study. **(B)** Microbial community composition from cellular fraction based on proteins summed abundances. **(C)** Microbial community composition from spent media based on protein counts. Each represented passage has 3 biological replicates. Each color in bar chart represents individual microbial member. **(D)** Percentage protein identified with the predicted secretion signal. Signal was predicted using SignalP 5.0 (http://www.cbs.dtu.dk/services/SignalP/).

**(a)**


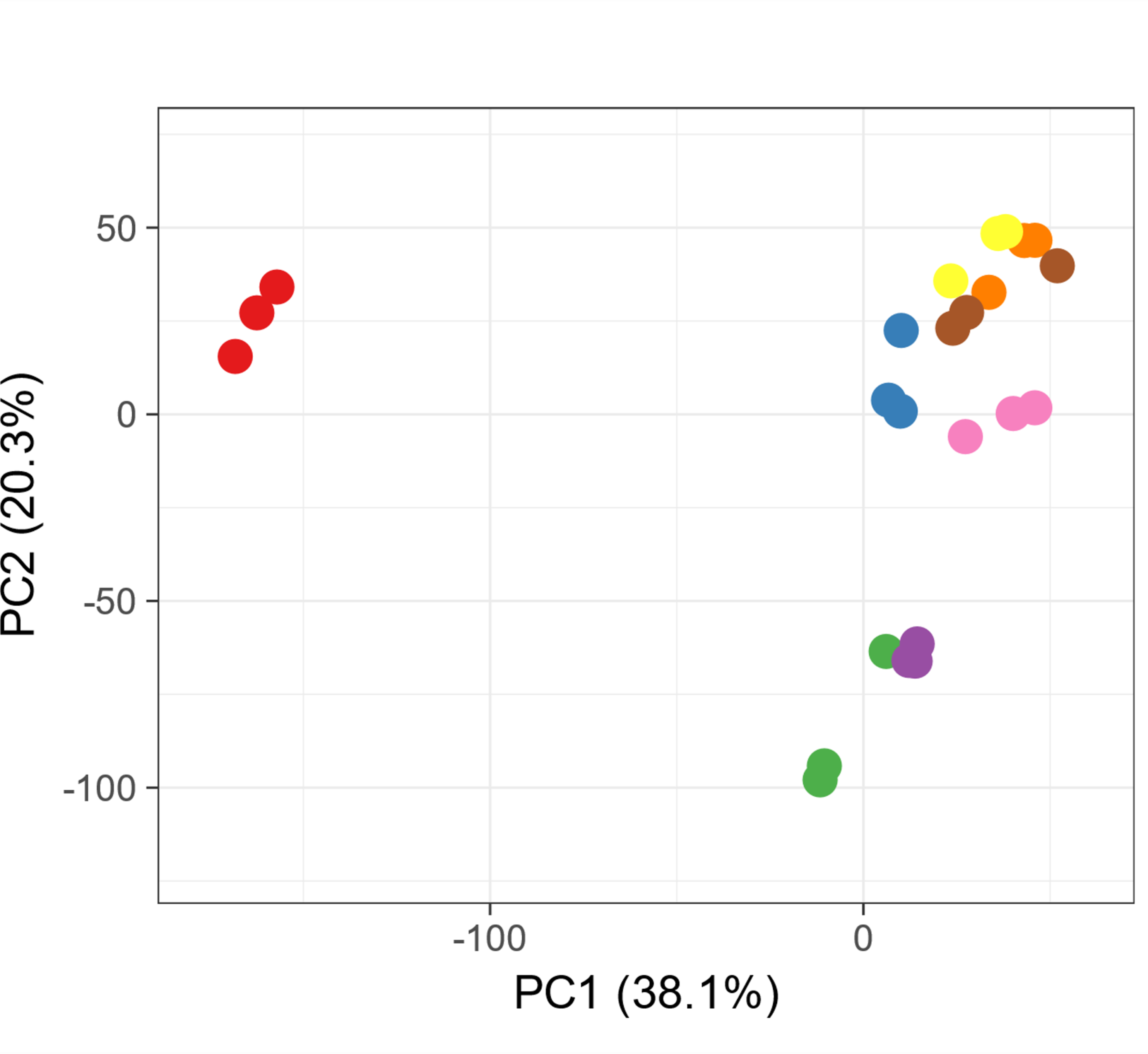

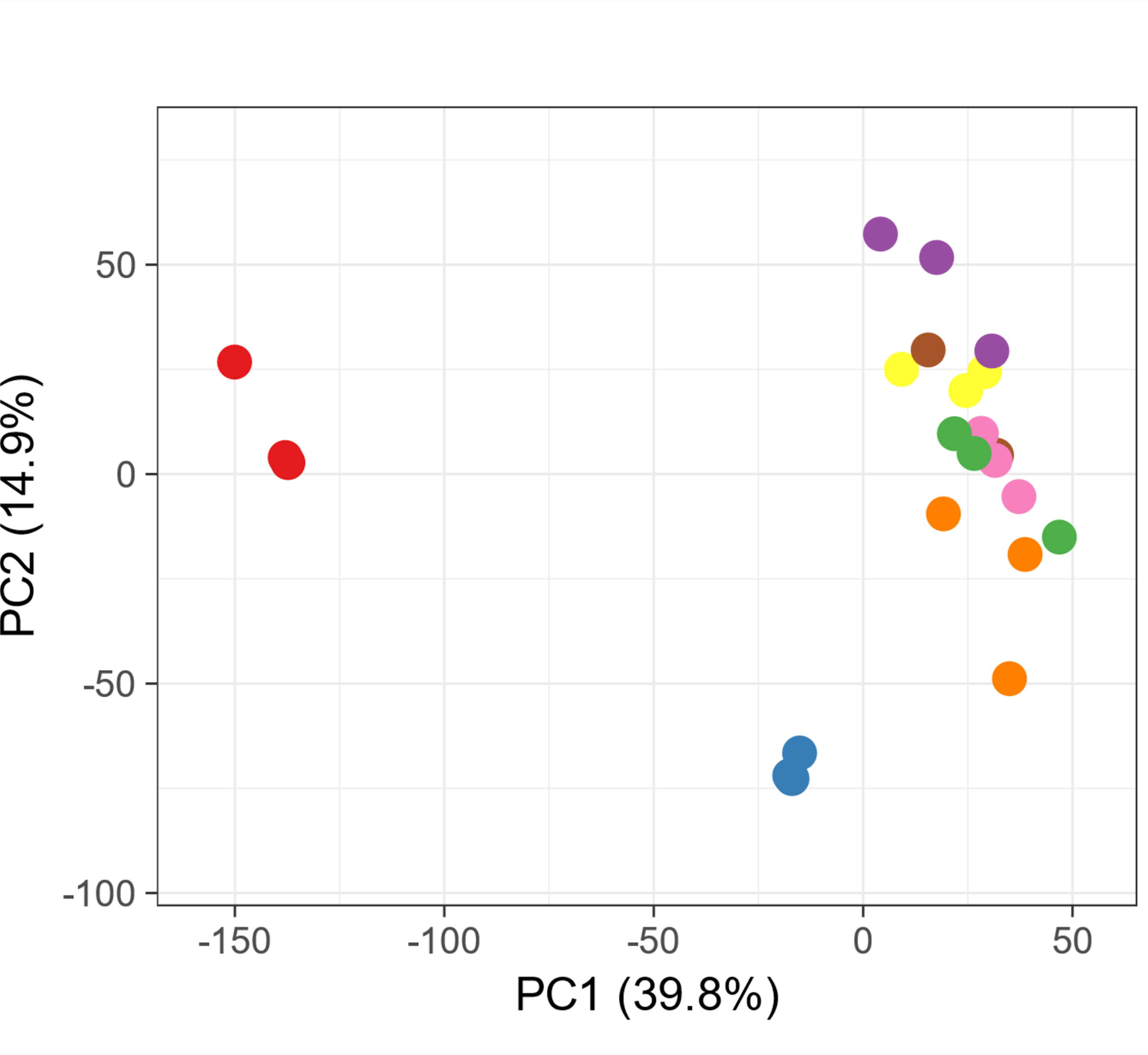

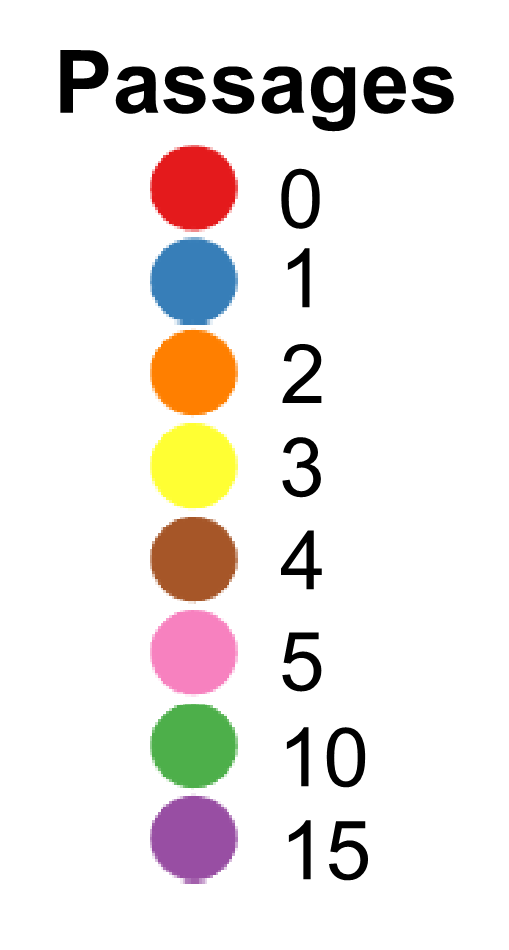

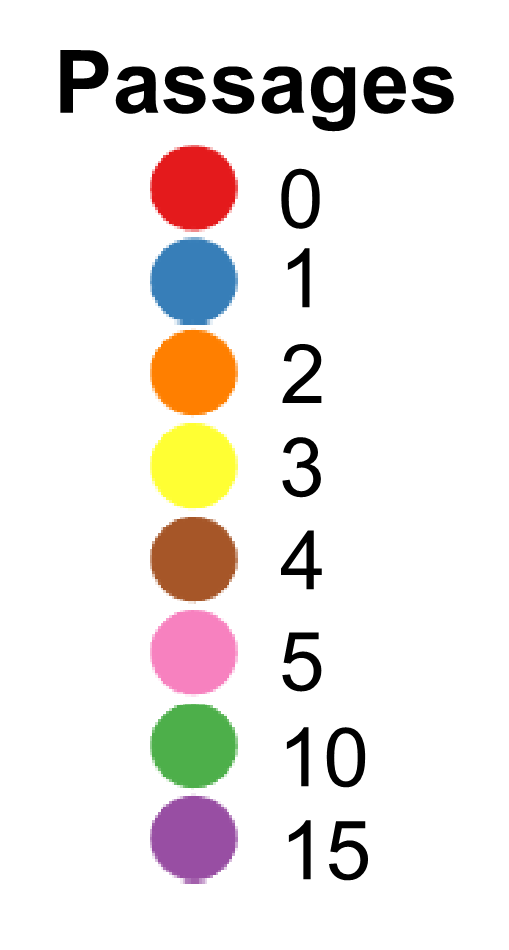


**(b)**

**Supplemental** **figure** **2:** **Principal** **component** **analysis** **(PCA)** **for** **visualizing** **clustering** **of** **multivariate** **data.** **(A)** PCA of samples from complex media. X and Y axis show principal component 1 and principal component 2 with the explain variance given in the percentage value. **(B)** PCA of samples from minimal media. X and Y axis show principal component 1 and principal component 2 with the explain variance given in the percentage value. ClustVis was utilized to generate the PCA plot.


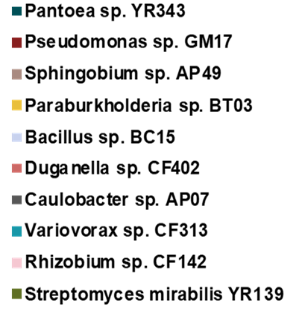


**Supplemental** **figure** **3:** **Significant** **proteins** **identified** **by student’s t-test between consecutive passages in both media.** The percent total of the number of significantly changing proteins per organism in MOPS (represented by M) media and R2A (represented by R) illustrates that *Pseudomonas* sp. GM17 in MOPS media and *Pseudomonas* sp. GM17, *Pantoea* sp. YR343 and *Bacillus* sp. BC15 in R2A are consistently present as top organisms with changing proteome profiles.

**
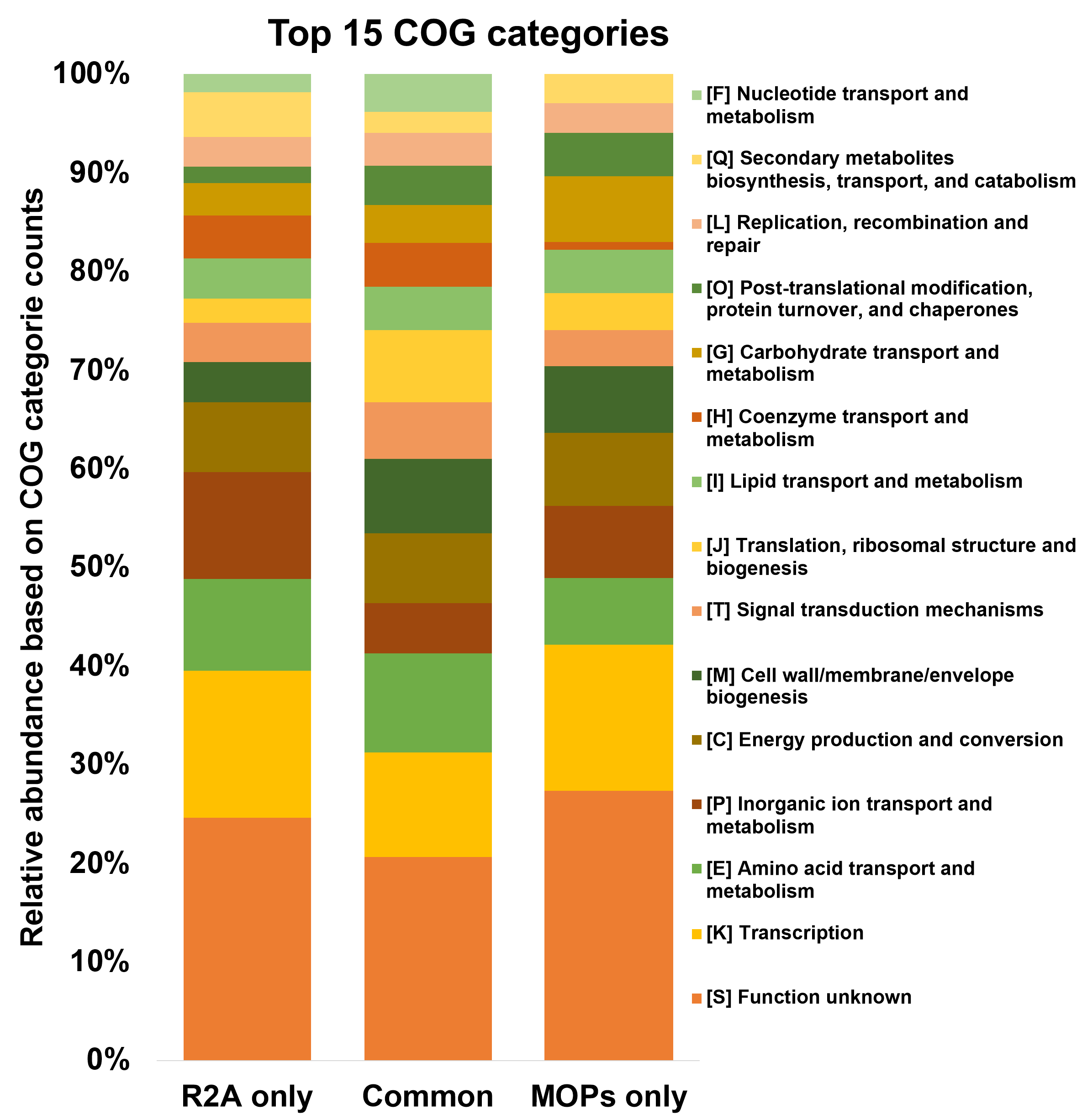
(a)**

**
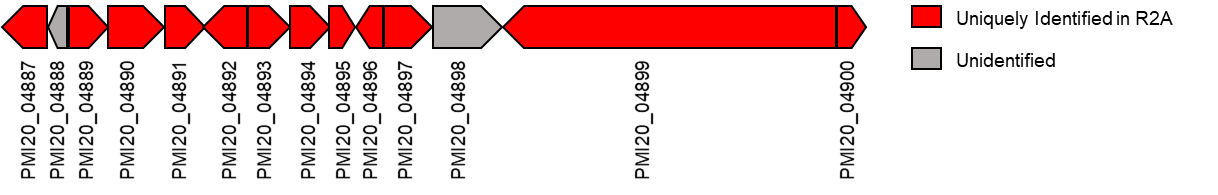
(b)**

**(c)**


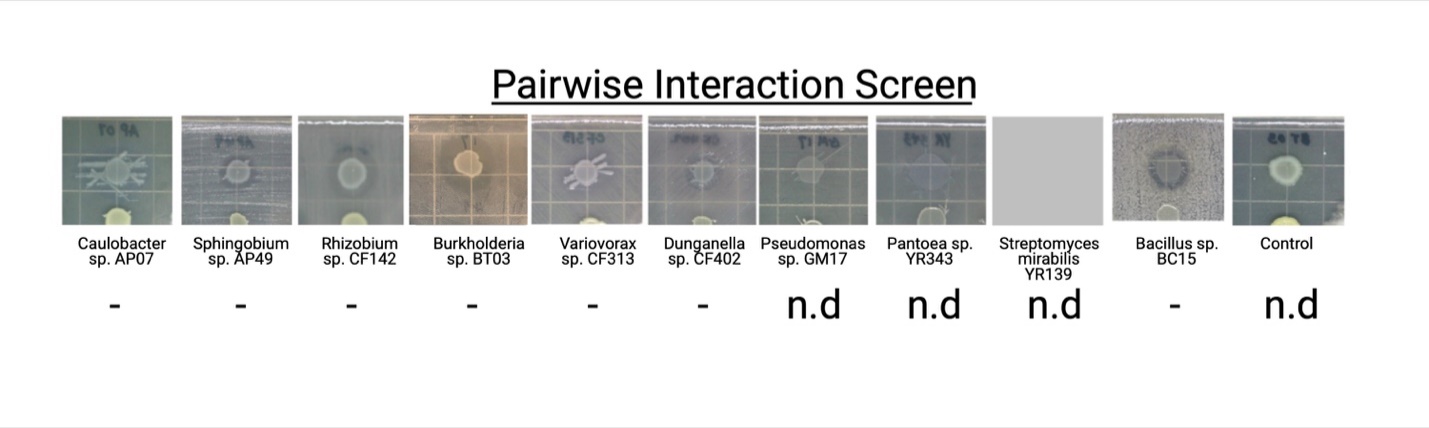
**Supplemental** **figure** **4: Evaluation of *Pseudomonas* sp. GM17 functional profiles and activity against individual community members. (A)** The stacked bar chart shows the top 15 COG categories from the proteins identified in R2A only, in both media and in MOPS only. **(B)** An example of uniquely identified NRPS pyoverdine BGC for *Pseudomonas* in R2A media. **(C)** *Pseudomonas* pairwise interaction screening result. The microbial lawn is prepared in agar plate using individual microbial members and *Pseudomonas* sp. GM17 was used for a colony in all the test conditions. The presence of a zone of inhibition (-) represents antagonistic interaction, no obvious phenotypic differences were represented by empty and the test where interaction couldn’t be determined were represented by n.d.


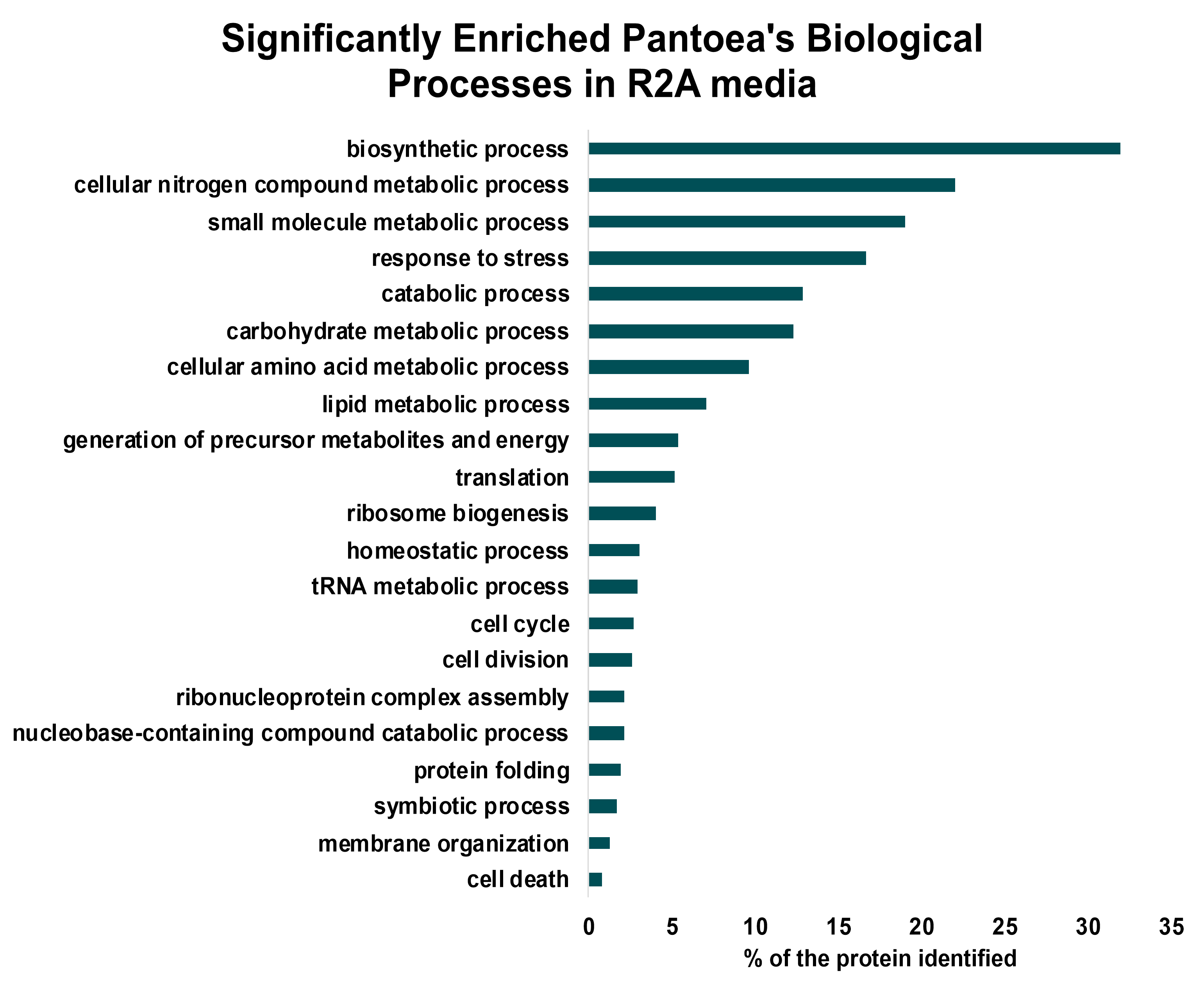

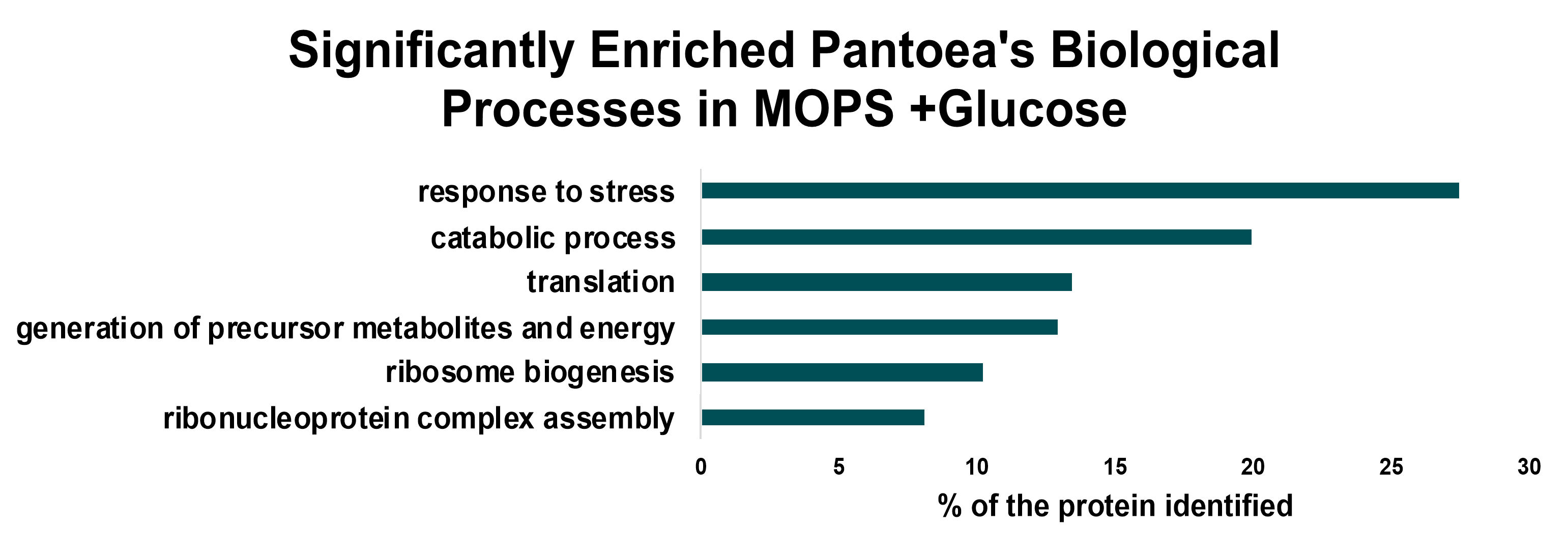


**Supplemental** **figure** **5:** **Functional** **level** **analysis** **of** ***Pantoea*** **proteome** **in** **complex** **and** **minimal** **media.** Bar chart shows the biological processes significantly enriched in complex and minimal media. Enrichment was carried out using gene-set analysis (GSA) in R library piano. The FDR threshold utilized was 5%.


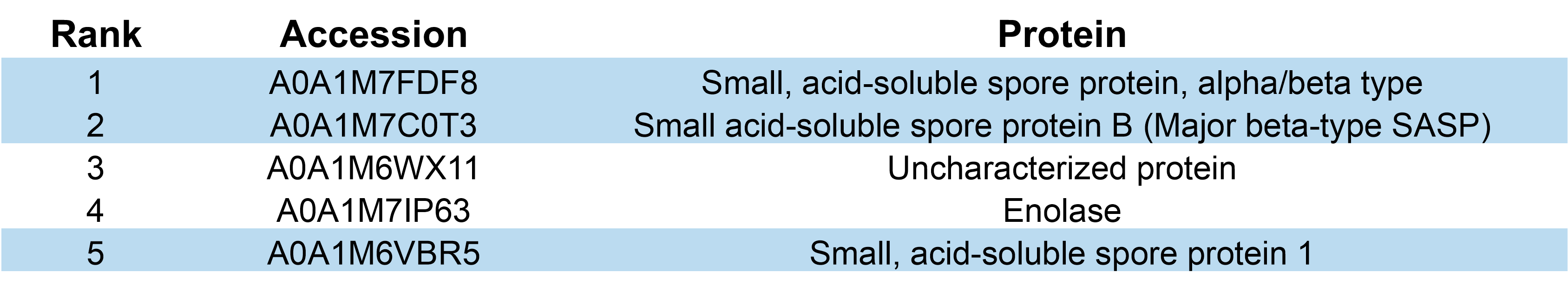

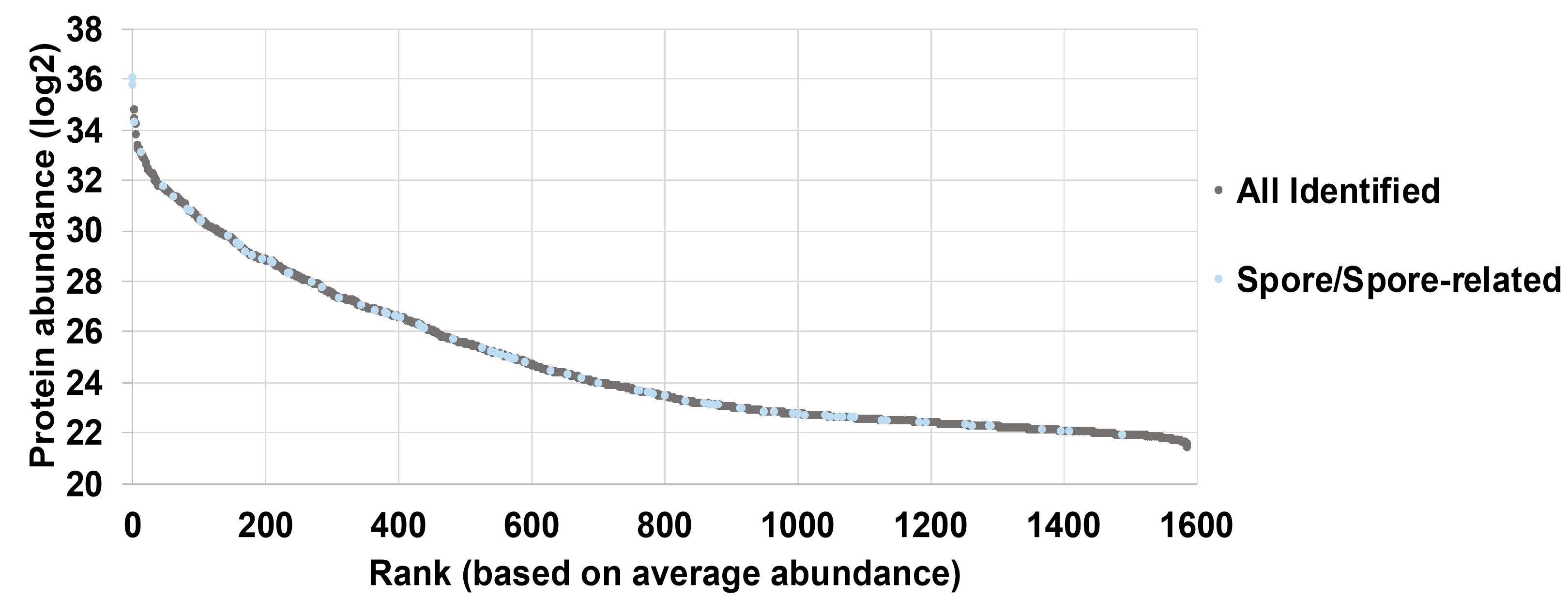


**Supplemental** **figure** **6:** **Spore** **and** **sporulation** **related** **proteins** **in** ***Bacillus*** **sp.** **BC15.** The rank-based distribution of *Bacillus* sp. BC15 proteins based on average abundance identified spore/spore-related proteins among the highly abundant. Table of top 5 proteins identified in *Bacillus* sp. BC15 shows 3 out of top 5 were spore proteins.


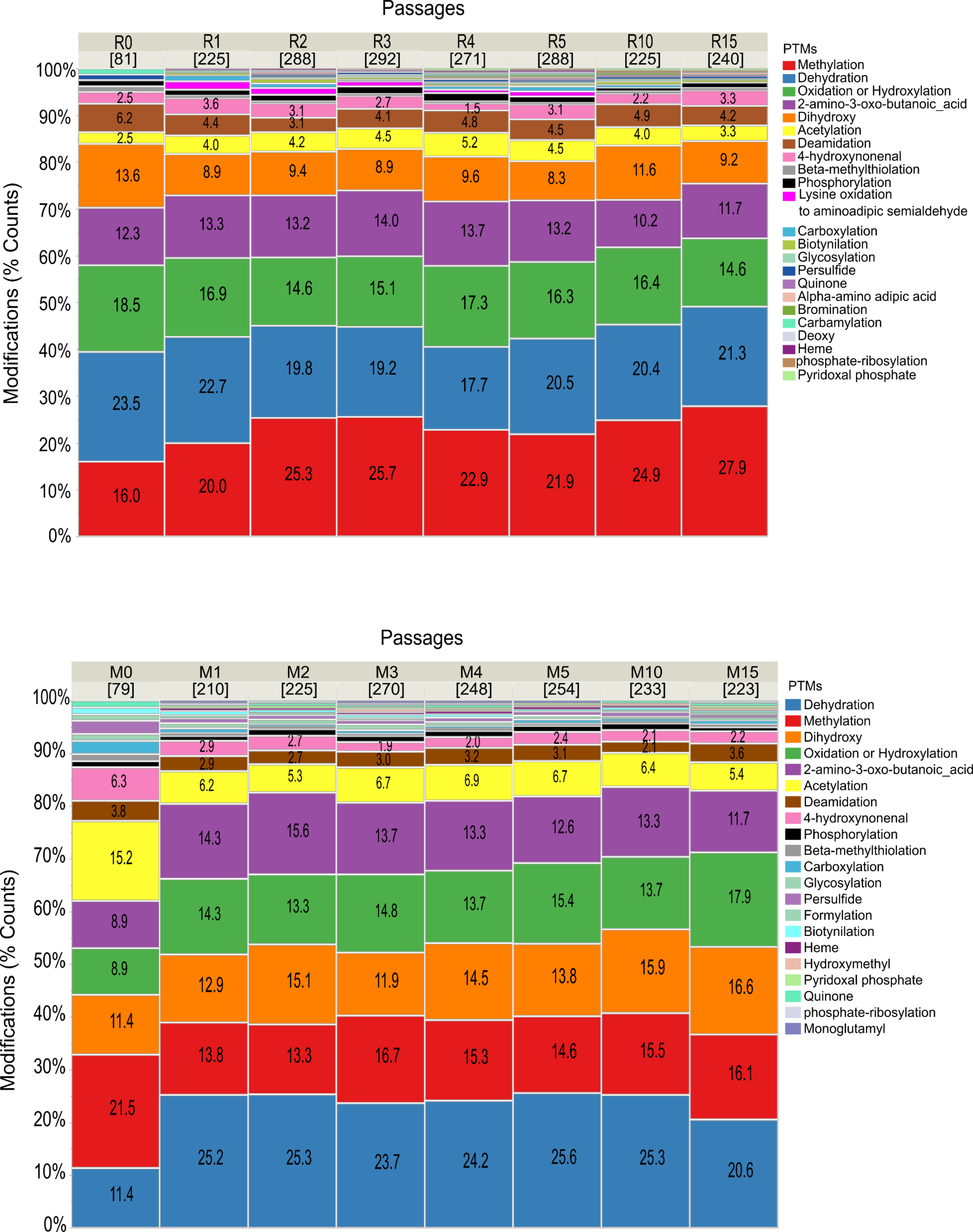
**(a)**

**(b)**

**Supplemental** **figure** **7.** **Percentage** **distribution** **of** ***Pseudomonas*** **proteins** **into** **classes** **of** **biologically** **relevant** **PTMs**. **(A)** Percentages of proteins into PTM categories identified in complex media conditions across the eight performed passages. **(B)** Percentages of proteins into PTM categories identified in minimal media conditions across the eight performed passages. Numbers in brackets are the total numbers of proteins from which the percentages were derived from. Proteins with more than one PTM are counted individually at each respective category.


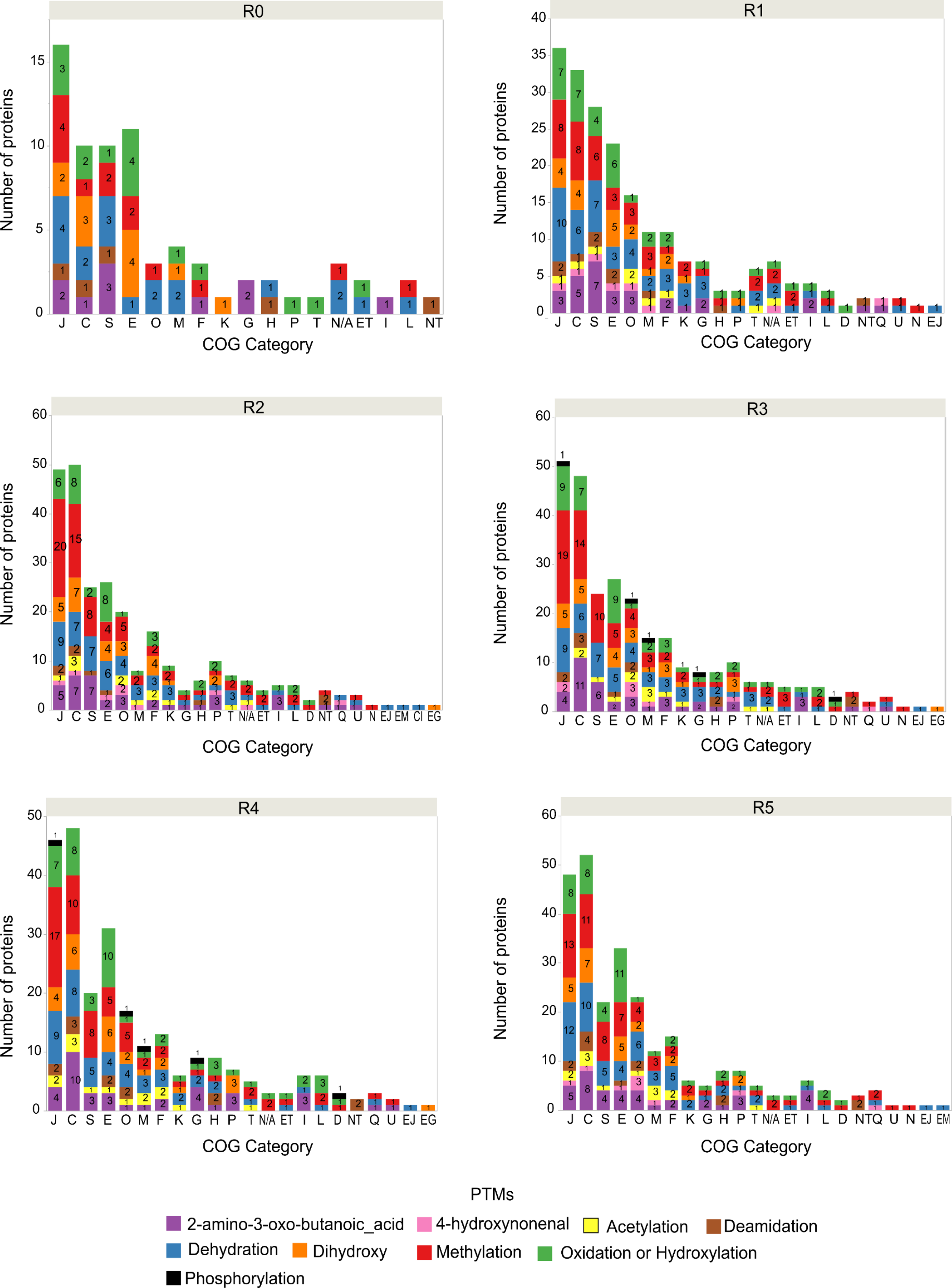

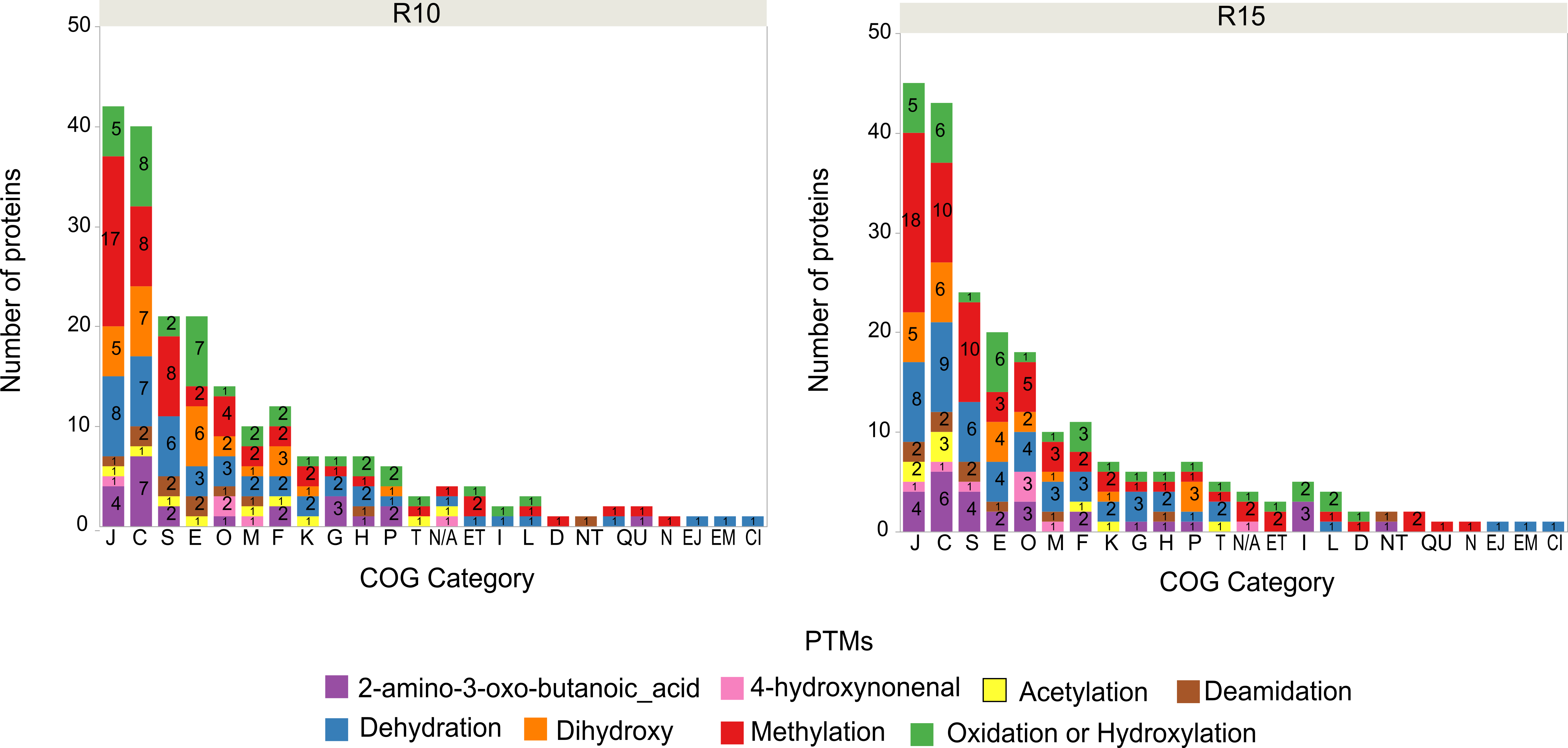


**Supplemental** **figure** **8.** **Distribution** **of** ***Pseudomonas*** **proteins** **with** **biologically** **relevant** **PTMs** **among** **COG** **categories** **by** **passage** **in** **complex** **media** **conditions**. For ease of representation, only PTM types with five or more proteins across COGs in a single passage are shown. Similar observations were obtained for *Pseudomonas* in minimal media. COG categories: A: RNA processing and modification; B: Chromatin Structure and dynamics; C Energy production and conversion; D: Cell cycle control, cell division, chromosome partitioning; E: Amino Acid metabolism and transport; F: Nucleotide metabolism and transport; G: Carbohydrate metabolism and transport; H: Coenzyme transport and metabolism; I: Lipid transport and metabolism; J: Translation, ribosomal structure and biogenesis; K: Transcription; L: Replication, recombination and repair; M: Cell wall/membrane/envelop biogenesis; N: Cell motility; O: Post-translational modification, protein turnover, chaperone functions; P: Inorganic ion transport and metabolism; Q: Secondary metabolites biosynthesis, transport, and catabolism; R: General Functional Prediction only; S: Function Unknown; T: Signal transduction mechanisms; U: Intracellular trafficking, secretion and vesicular transport; Y: Nuclear structure; Z: Cytoskeleton. Proteins annotated with more than one category are also shown. N/A, No COG category assigned.


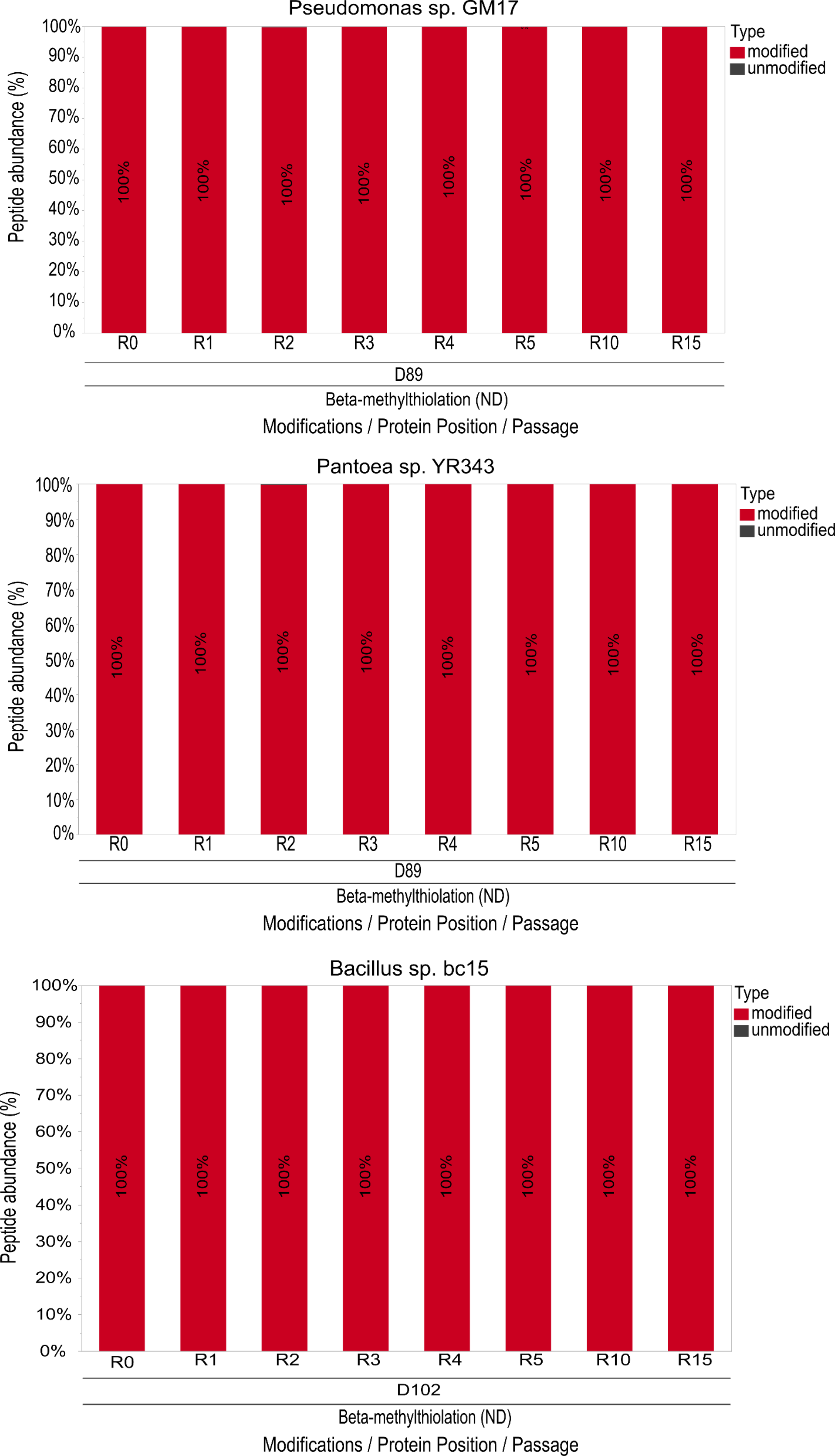


**Supplemental** **figure** **9.** **Beta-methylthiolation** **of** **aspartic** **acid** **residues** **in** **ribosomal** **proteins** **S12** **in** **complex** **media** **conditions**. Percentage abundances of beta-methylthiolated Asp modified and unmodified peptides in the ribosomal S12 proteins of *Pseudomonas*, *Pantoea* and *Bacillus* in complex media, respectively. Similar results were observed for *Pseudomonas* at minimal media conditions, and for other less abundant microorganisms.
