# Supplementary material for: Metaproteomics reveals insights into microbial structure, interactions, and dynamic regulation in defined communities as they respond to environmental disturbance
Source: BMC Microbiol. 2021 Nov 8;21:308. doi: 10.1186/s12866-021-02370-4 (PMC8574000; doi:10.1186/s12866-021-02370-4)
Supplement: Supplementary file 9 — Additional file 9. [file 12866_2021_2370_MOESM9_ESM.docx]

**Table 8**. List of proteins in which the percentage abundance of modified to unmodified peptides is ≥ 90% across at least 6/8 passages in either rich or minimal media conditions.

| **Organism** | **Datasets** | **Protein (Accession)** | **Modification & AA position in protein** |
| --- | --- | --- | --- |
| *Pseudomonas* | Rich | Universal stress protein (WP_007921319) | Acetylation K76 |
| *Pseudomonas* | Rich | Ribosomal S12 (WP_003186084.1) | Beta-methylthiolation D89 |
| *Pseudomonas* | Rich | DUF1254 domain-containing protein (WP_007923239.1) | Carboxylation E300 |
| *Pseudomonas* | Rich | Methyl-accepting chemotaxis protein (WP_007930907) | Deamidation followed by a methylation Q517 |
| *Pseudomonas* | Rich | 50S ribosomal protein L7/L12 | Dimethylation K82 |
| *Pseudomonas* | Rich | Electron transfer flavoprotein subunit beta/Fix A family protein (WP_007928126.1) | Dimethylation K196 |
| *Pseudomonas* | Rich | DnaJ domain-containing protein (WP_007923673.1) | Methylation R274 |
| *Pseudomonas* | Rich | MULTISPECIES: 30S ribosomal protein S1 (WP_007931318.1) | Methylation K119 |
| *Pseudomonas* | Rich | 50S Ribosomal protein L3 (WP_007924205.1) | Methylation T151 |
| *Pseudomonas* | Rich | MULTISPECIES: cytochrome c4 (WP_009041529.1) | Oxidation or Hydroxylation Y59 |
| *Bacillus* | Rich | 30S ribosomal protein S12 (A0A1M7LX31) | Beta-methylthiolation D102 |
| *Bacillus* | Rich | 50S ribosomal protein L7/L12 (A0A1M7LV54) | Dimethylation K80 |
| *Bacillus* | Rich | Elongation factor Tu (A0A1M7LVX3) | Oxidation or Hydroxylation P54 |
| *Pantoea* | Rich | 30S ribosomal protein S12 (J3HR48) | Beta-methylthiolation D89 |
| *Pantoea* | Rich | Elongation factor Tu (J3HLV4) | Dimethylation K14 |
| *Pantoea* | Rich | 50S ribosomal protein L7/L12 (J3HFH7) | Methylation K82 |
| *Pantoea* | Rich | 50S ribosomal protein L3 (J2V1V3) | Methylation T150 |
| *Duganella* | Rich | Ribonuclease R (A0A1H7KA63) | Dimethylation (KR) R779 |
| *Duganella* | Rich | Bifunctional protein PutA (A0A1H8CPF2) | Methylation (KR) R116 |
| *Burkholderia* | Rich | arginine deiminase (WP_007738396.1) | Carboxylation E351 |
| *Rhizobium* | Rich | Elongation factor Tu (J1SLR3) | Dimethylation (KR) R54 |
| *Variovorax* | Rich | ATP synthase subunit beta (J2TIC3) | Dehydration S35 |
| *Pseudomonas* | Minimum | MULTISPECIES: 30S ribosomal protein S3 (WP_007924192.1) | Acetylation K80 |
| *Pseudomonas* | Minimum | MULTISPECIES: 30S ribosomal protein S12 (WP_003186084.1) | Beta-methylthiolation D89 |
| *Pseudomonas* | Minimum | MULTISPECIES: potassium channel family protein (WP_007920975.1) | Carboxylation E190 |
| *Pseudomonas* | Minimum | MULTISPECIES: methyl-accepting chemotaxis protein (WP_007924881.1) | Deamidation followed by a methylation Q518 |
| *Pseudomonas* | Minimum | MULTISPECIES: 23S rRNA (uracil(1939)-C(5))-methyltransferase RlmD | Dehydration S101 |
| *Pseudomonas* | Minimum | MULTISPECIES: 50S ribosomal protein L7/L12 (WP_007925956.1) | Dimethylation(KR) K82 |
| *Pseudomonas* | Minimum | MULTISPECIES: electron transfer flavoprotein subunit beta/FixA family protein (WP_007928126.1) | Dimethylation(KR) K196 |
| *Pseudomonas* | Minimum | MULTISPECIES: 30S ribosomal protein S1 (WP_007931318.1) | Methylation(KR) K119 |
| *Pseudomonas* | Minimum | MULTISPECIES: DNA binding protein (WP_007931174.1) | Methylation(KR) R52 |
| *Pseudomonas* | Minimum | MULTISPECIES: DUF971 domain-containing protein (WP_007925870.1) | Methylation(KR) K122 |
| *Pseudomonas* | Minimum | MULTISPECIES: 50S ribosomal protein L3 (WP_007924205.1) | Methylation T151 |
| *Pseudomonas* | Minimum | MULTISPECIES: preprotein translocase subunit SecA (WP_007931158.1) | Methylation (Protein N-term) M1 |
| *Pseudomonas* | Minimum | MULTISPECIES: cytochrome c4 (WP_009041529.1) | Oxidation/Hydroxylation Y59 |
| *Bacillus* | Minimum | Polyribonucleotide nucleotidyltransferase (A0A1M7GJR9) | Formylation (Protein N-term) (Q6) |
| *Duganella* | Minimum | Ribonuclease R (A0A1H7KA63) | Dimethylation (KR) R779 |
| *Pantoea* |  | 30S ribosomal protein S3 (J3HLV0) | Acetylation (N-term) K80 |
| *Rhizobium* | Minimum | 30S ribosomal protein S12 (J2B0B0) | Beta-methylthiolation (ND) D89 |
| *Rhizobium* | Minimum | Elongation factor Tu (Fragment) | Dimethylation(KR) R54 |
